# A multi-objective optimization consensus model for large-scale group decision-making considering dynamic social networks

**DOI:** 10.1038/s41598-026-45239-0

**Published:** 2026-04-01

**Authors:** Gang Chen, Anqiong Lang, Xun Han, Jiangyue Fu, Xinchuan Liu

**Affiliations:** 1 School of Management, Guizhou University, Guiyang, 550025 China; 2 Digital Transformation and Governance Innovation Laboratory, Guizhou University, Guiyang, 550025 China; 3 Intelligent Policing Key Laboratory of Sichuan Province, Sichuan Police College, Luzhou, 646000 China; 4 Department of Transportation Management, Sichuan Police College, Luzhou, 646000 China; 5 Department of Engineering and Industrial Professions, University of North Alabama, Florence, USA

**Keywords:** Large-scale group decision-making, Multi-objective optimization, Consensus reaching process, Dynamic trust networks, Energy science and technology, Engineering, Mathematics and computing

## Abstract

As global climate change intensifies and fossil fuel resources continue to deplete, transitioning to alternative energy sources has become a vital strategy for sustainable development. Photovoltaic (PV) power generation, recognized for its clean, renewable, and low-carbon characteristics, is advancing rapidly. Against this background, site selection for PV power plant stations has become a crucial decision-making factor in ensuring project success. However, in large-scale group decision-making (LGDM), the complex backgrounds and substantial number of decision-makers (DMs) pose a significant challenge in reaching consensus efficiently. Therefore, this paper proposes a multi-objective optimization consensus model (MOOCM) utilizing dynamic trust networks to solve LGDM problems. First, a hybrid trust network (HTN) is built by integrating preference similarity and trust relationships, and DMs are clustered using the Louvain algorithm based on this hybrid network. Second, a MOOCM is designed with the objectives of minimizing costs, maximizing fairness, and achieving a high consensus level. Then, after consensus is reached, the HTN is updated, and secondary clustering is performed to obtain dynamic weights for DMs. Finally, a PV power plant site selection problem with 20 DMs, four alternatives, and four attributes is used as a case study for validation. In the first clustering, the DMs are divided into four subgroups. After consensus is reached, the HTN is updated and a second clustering is performed, which finally produces three subgroups. At the same time, the proposed method can achieve a group consensus level (GCL) of 0.9597, with Cost = 1.7422 and Fairness = 0.9043. These results verify the effectiveness and practical utility of the proposed method in LGDM.

**Supplementary Information:**

The online version contains supplementary material available at 10.1038/s41598-026-45239-0.

## Introduction

As global climate change becomes increasingly severe and fossil fuels become depleted, switching to new energy sources has become a shared strategic goal for nations to achieve sustainable development. The heavy use of traditional fossil fuels, especially oil and coal^1^, has made global warming worse. This environmental pressure is pushing many countries to move more quickly toward new energy systems, such as solar, wind, and geothermal power^2^. It is estimated that by 2050, solar and wind power will make up 52% of the total electricity generation^3^. Solar energy, in particular, has become the most popular renewable energy source due to its clean, renewable, and low-carbon advantages^4^.

Therefore, to ensure more effective photovoltaic (PV) power plant construction, selecting suitable locations based on regional solar resources and energy demand differences has become a key issue. Wu et al.^5^ proposed a multi-criteria decision framework, combining DEMATEL-TODIM, to select PV power plant locations in highway service areas. Demir et al.^6^ designed a PV site selection model applying GIS together with multi-criteria analysis. Wan et al.^2^ developed a heterogeneous multi-attribute group decision-making (GDM) approach to determine the optimal locations for PV power plants. Zhang and Zhao^7^ introduced a hybrid multi-criteria decision-making approach built on resource-demand matching to evaluate and select the best PV power plant site options.

In GDM, several decision-makers (DMs) participate in evaluating alternatives and forming a collective preference by combining their individual opinions to select the best option^8^. The goal of GDM is to address unstructured decision problems^9^. Bilal et al.^10^ developed a novel aggregation-driven GDM algorithm to systematically integrate diverse expert evaluations, thereby ensuring a consistent ranking of alternatives. Khan et al.^11^ proposed a new class of aggregation operators to handle uncertain and imprecise information in multi-attribute GDM. As information technology leaps and bounds, traditional GDM is progressively transforming into large-scale group decision-making (LGDM)^12^. LGDM usually involves more than 20 decision participants^13^ and includes processes such as clustering, consensus measurement, feedback mechanisms, and alternative selection.

LGDM usually includes many participants from varied professional fields. To simplify the decision process, DMs are divided into subgroups. Clustering approaches are typically categorized into two types. One is to group DMs based on distance and preference similarity. The other is combined with social network analysis and clusters individuals according to trust relationships. Yu et al.^14^ set boundary constraints on subgroup sizes for DMs and developed a clustering approach based on distance. Wu et al.^15^ further suggested an improved K-means clustering algorithm that combines preferences with their modification costs. However, classifying DMs without trust relationships into the same subgroup merely considering preference similarity, it may not be conducive to consensus reaching. To tackle this issue, Qin et al.^16^ considered trust relationships and introduced a Louvain algorithm based on multi-level modularity optimization. Meng et al.^17^ further clustered DMs by integrating preference similarity with trust. In general, most clustering approaches mainly relies on a single factor, either preferences or trust, and limited studies have addressed both factors simultaneously.

Different DMs have their own preferences, which may lead to differences and conflicts in opinions. A feedback mechanism is commonly utilized during the consensus reaching process (CRP) to facilitate achieving agreement. This mechanism is adopted to promote maximum agreement among DMs and to guarantee that the final outcome gains approval from most participants^18,19^. Feedback mechanisms can be categorized into two primary types. The first type relies on identification and guidance rules^20^, which firstly identifies DMs who need to adjust their preferences and provide them with suggestions for adjustment. Li et al.^21^ developed a consensus measurement method to enhance the consensus level by identifying DMs who need to adjust their preferences and suggesting modifications. The second type is founded on optimization models^17,22^, which intend to control the extent of modifications while reducing their costs. Wu et al.^23^ designed a dual personalized feedback mechanism that uses personalized feedback acceptance coefficients to reach consensus while minimizing the total adjustment cost. Liu et al.^24^ constructed an optimization model aimed at maximizing satisfaction and proposed a satisfaction-oriented two-stage feedback mechanism to address non-cooperative behaviors. The first type of feedback mechanism often requires many iterations and high time costs, while the second type overcomes these drawbacks. Yet existing studies have not fully considered fairness during the feedback adjustment process or how to maximize the consensus level.

In actual decision-making processes (DMP), the interactions among DMs may lead to changes in their trust relationships^25^. In essence, DMs tend to be more affected by those they trust^26^. Teng et al.^27^developed a dynamic weighting approach that integrates trust relationships with evidential conflicts. Xing et al.^25^ updated trust relationships according to changes in the consensus state after each iteration round. Peng et al.^28^ updated the trust network using historical data and established a hybrid framework that integrates preference similarity with trust relationships. Clearly, trust relationships are not static, and greater attention should be paid to how interactions among DMs lead to changes in trust. However, the studies mentioned above do not consider that after reaching a consensus, the degree of trust among some DMs may increase due to higher opinion similarity with others. Therefore, by updating the trust network, the weights assigned to each DM can be made more reasonable, leading to a more rational final decision outcome.

Based on the above analysis, LGDM has been widely studied; nevertheless, the current literature still exhibits several limitations, which are summarized and compared with our work in Table 1. Most current clustering methods consider only trust relationships or preference similarity and rarely account for the influence of both factors. Similarly, optimization models have paid limited attention to both fairness and maximizing the consensus level. Additionally, there has been limited research on updating trust networks, re-clustering, and adjusting DMs’ weights in the post-CRP operation phase.

**Table 1:** Summary and comparison of related LGDM studies.

Reference	Clustering basis	Feedback mechanism	Considered fairness	Considered maximization the GCL	Dynamic trust updating	Re-clustering / weight adjustment
Yu et al.^14^	Preference distance	Rule-based	—	—	—	—
Wu et al.^15^	Preference similarity + adjustment cost	Optimization-based	×	×	—	—
Qin et al.^16^	Trust relationships	Optimization-based	×	×	—	—
Meng et al.^17^	Preference similarity + trust relationships	Optimization-based	×	×	√	×
Zheng et al.^18^	Preference similarity and compatibility	Optimization-based	×	×	—	—
Wu et al.^19^	Preference similarity	Rule-based	—	—	—	Weight adjustment
Peng et al.^20^	Trust relationships	Rule-based	—	—	—	—
Li et al.^21^	—	Rule-based	—	—	—	—
Guo et al.^22^	—	Optimization-based	×	×	—	—
Wu et al.^23^	—	Optimization-based	×	×	—	—
Liu et al.^24^	—	Optimization-based	×	×	—	—
Xing et al.^25^	—	Rule-based	—	—	√	×
Liu et al.^26^	—	Rule-based	—	—	√	Weight adjustment
Teng et al.^27^	—	Optimization-based	×	×	√	×
Peng et al.^28^	Preference similarity + trust relationships	Rule-based	—	—	√	Weight adjustment
This study	Preference similarity + trust relationships	Optimization-based	√	√	√	√

“√” indicates that the corresponding aspect is explicitly considered in the study; “×” indicates not considered; “—” indicates not applicable or not reported in the study.

Given the issues discussed above, this paper proposes a multi-objective optimization consensus model (MOOCM) for LGDM in dynamic trust networks. The key contributions are outlined as follows:

1. A Louvain clustering method based on a hybrid trust network (HTN) is proposed. By combining preference similarity and trust relationships to construct the HTN, the method effectively divides DMs. This reduces the complexity of LGDM and improves the rationality of the clustering results.
2. A MOOCM is proposed, driven by the goals of minimizing cost, maximizing fairness, and maximizing the group consensus level (GCL). This model can coordinate the trade-offs among cost, fairness, and GCL at the same time. It also generates a Pareto optimal solution set, which provides several compromise options for practical DMP and can be flexibly applied to different decision scenarios.
3. A dynamic trust network updating mechanism is proposed. During the feedback adjustment process, DMs’ preferences change through interactions, and preference similarity then affects the trust. After consensus is reached, the preference similarity is recalculated based on the adjusted preferences, and the HTN is dynamically updated. Then, a second clustering is conducted to dynamically adjust the weights of DMs, which can more accurately describe the evolution of trust relationships.

The structure of this paper is as follows. Section 2 introduces the fundamental concepts of uncertain linguistic variables, social network analysis (SNA), the Louvain algorithm, and optimization models. Section 3 constructs a CRP for LGDM in dynamic trust networks. Section 4 uses a case study to demonstrate the effectiveness of the proposed method. Section 5 presents a sensitivity analysis and a comparative analysis. Finally, Sect. 6 summarizes the conclusions and outlines potential directions for future research.

## Preliminaries

### Uncertain linguistic variables

In daily life, people often convey their evaluations by qualitative natural language expressions, such as linguistic terms. Using linguistic terms to evaluate alternatives helps reflect DMs’ preferences^29^. However, the complexity of decision environments and the lack of sufficient knowledge or expertise may cause random errors in preference data, which often leads to the uncertainty of preferences information^30^. In practice, a DM might consider an alternative to be somewhere between “good” and “very good.” At this point, introducing uncertain linguistic variables can help them express their preferences more flexibly.

Let ${\boldsymbol{S}}=\left\{ {{s_\varsigma }|\varsigma =0,1, \ldots ,q} \right\}$ be a set of linguistic terms, and ${s_\varsigma }$ be one of these terms. Single linguistic terms often struggle to fully express a DM’s true preferences. Therefore, this paper adopts interval values composed of two linguistic terms as preference information to evaluate alternatives.

#### **Definition 1**^31^

Let $\tilde {s}=\left[ {{s_L},{s_U}} \right]$ be an uncertain linguistic variable, where ${s_L}$ and ${s_U}$ are the lower and upper bounds of $\tilde {s}$, and ${s_L},{s_U} \in {\boldsymbol{S}}$. Let ${\boldsymbol{S}}$ be a set of uncertain linguistic variables. Suppose ${\tilde {s}_1}=\left[ {{s_{{L_1}}},{s_{{U_1}}}} \right]$ and ${\tilde {s}_2}=\left[ {{s_{{L_2}}},{s_{{U_2}}}} \right]$ are any two uncertain linguistic variables in ${\boldsymbol{S}}$. The algorithms of these variables are specified as follows:

1. $$\tilde{s}_{1} \oplus \tilde{s}_{2} = \left[ {s_{{L_{1} }} \oplus s_{{L_{2} }} {,}s_{{U_{1} }} \oplus s_{{U_{2} }} } \right] = \left[ {s_{{L_{1} + L_{2} }} {,}s_{{U_{1} + U_{2} }} } \right]$$
2. $$\vartheta {\tilde {s}_1}=\left[ {\vartheta {s_{{L_1}}},\vartheta {s_{{U_1}}}} \right]=\left[ {{s_{\vartheta {L_1}}},{s_{\vartheta {U_1}}}} \right]$$, where $$\vartheta \in \left[ {0,1} \right]$$;
3. $$\vartheta \left( {{{\tilde {s}}_1} \oplus {{\tilde {s}}_2}} \right)=\vartheta {\tilde {s}_1} \oplus \vartheta {\tilde {s}_2}$$, where $$\vartheta \in \left[ {0,1} \right]$$;
4. $$\left( {{\vartheta _1}+{\vartheta _2}} \right){\tilde {s}_1}={\vartheta _1}{\tilde {s}_1} \oplus {\vartheta _2}{\tilde {s}_1}$$, where $${\vartheta _1},{\vartheta _2} \in \left[ {0,1} \right]$$.

#### Definition 2

The distance between any two uncertain linguistic variables $\tilde {s}_{1}^{{}}$ and $\tilde {s}_{2}^{{}}$ is defined as:

1 $$d\left( {{{\tilde {s}}_1},{{\tilde {s}}_2}} \right)=\frac{{\left| {f\left( {{s_{{L_1}}}} \right) - f\left( {{s_{{L_2}}}} \right)} \right|+\left| {f\left( {{s_{{U_1}}}} \right) - f\left( {{s_{{U_2}}}} \right)} \right|}}{{2q}}$$

where $f\left( {{s_{{L_1}}}} \right)$ represents the subscript function of the lower bound of the uncertain linguistic variable ${\tilde {s}_1}$. For example, if ${s_{{L_1}}}$ is a linguistic term with index ${L_1}$, then $f\left( {{s_{{L_1}}}} \right)={L_1}$.

#### Definition 3

Two uncertain linguistic variables can be compared using a dominance index (DI). Specifically, if ${s_L}$ and ${s_U}$ are farther from ${s_0}$and closer to ${s_q}$, it indicates that $\tilde {s}$ is superior. Therefore, the dominance index of $\tilde {s}$ is defined as:

2 $$DI\left( {\tilde {s}} \right)=\frac{{\sqrt {{{\left[ {f\left( {{s_L}} \right)} \right]}^2}+{{\left[ {f\left( {{s_U}} \right)} \right]}^2}} }}{{\sqrt {{{\left[ {f\left( {{s_L}} \right) - q} \right]}^2}+{{\left[ {f\left( {{s_U}} \right) - q} \right]}^2}} +\sqrt {{{\left[ {f\left( {{s_L}} \right)} \right]}^2}+{{\left[ {f\left( {{s_U}} \right)} \right]}^2}} }}$$

### Social network analysis

Social network analysis (SNA) is a method that studies the relationships between social entities, such as organizations, companies, or countries^32–34^. This approach has been extensively used in multiple fields, including GDM^35^, behavior analysis^36^, and multi-agent systems^37^.

SNA consists of three key components: the set of DMs, the relationships among DMs, and the attributes of DMs^38^. Social networks are commonly represented in three forms: graphs, matrices, or algebra^39^, as shown in Table 2.

**Table 2:** Three common representations of social network.

Graph	Matrix	Algebra
	$${\boldsymbol{A}}=\left[ {\begin{array}{*{20}{c}} 0&0&1 \\ 1&1&0 \\ 0&1&0 \end{array}} \right]$$	${v_1}R{v_3}$, ${v_2}R{v_3}$, ${v_1}R{v_4}$, ${v_4}R{v_2}$

#### **Definition 4**^26^

Suppose a social network is defined as a directed graph $G=\left( {V,E} \right)$, where ${\boldsymbol{V}}=\left\{ {{v_e}|e=1,2,...,k} \right\}$ represents the node set, and *E* consists of directed edges indicating the trust relationships among DMs.

#### Definition 5^40^

In the $G=\left( {V,E} \right)$, ${\boldsymbol{A}}={\left( {{a_{eh}}} \right)_{k \times k}}$ is the 0–1 adjacency matrix. If there is a directed edge from node ${v_e}$ to ${v_h}$, then ${a_{eh}}=1$; otherwise, ${a_{eh}}=0$, as shown in Eq. (3):

3 $${a_{eh}}=\left\{ \begin{gathered} 0, \left( {{v_e},{v_h}} \right) \notin E \hfill \\ 1, \left( {{v_e},{v_h}} \right) \in E \hfill \\ \end{gathered} \right.$$

#### **Definition 6**^41^

Suppose $\lambda =\left( {t,d} \right)$ is a two-dimensional vector, where *t* is the degree of trust and *d* is the degree of distrust, and it satisfies $t,d \in \left[ {0,1} \right]$. It is called the trust function. Wu et al.^41^ defined the trust score as:

4 $$TS\left( \lambda \right)=\frac{{t - d+1}}{2}$$

where $0 \leqslant TS\left( \lambda \right) \leqslant 1$. The trust score represents the degree of normalized dominance of the trust value relative to the distrust value in the trust function.

#### **Definition 7**^39^

Let ${\boldsymbol{T}}{\boldsymbol{M}}={\left( {\left( {{t_{eh}},{d_{eh}}} \right)} \right)_{k \times k}}$ be a social network matrix, where $\left( {{t_{eh}},{d_{eh}}} \right)$ represents the trust function from node ${v_e}$ to ${v_h}$, then the trust score of node ${v_h}$ is:

5 $$T{S_h}=\frac{1}{{k - 1}} \times \sum\limits_{{e=1,e \ne h}}^{k} {\frac{{{t_{eh}} - {d_{eh}}+1}}{2}}$$

where $T{S_h} \in \left[ {0,1} \right]$, and $T{S_h}$ represents the average trust score of other decision makers towards DM ${v_h}$. The social network matrix is then denoted as ${\boldsymbol{T}}{\boldsymbol{M}}={\left( {T{S_{eh}}} \right)_{k \times k}}$.

### Louvain algorithm

The Louvain algorithm is a community detection method that relies on optimizing modularity^42^. It is essentially a greedy algorithm, and its core idea is to move nodes to the community where their neighboring nodes are located and select the neighboring community with the largest modularity gain until the modularity no longer increases. This algorithm not only maintains high clustering quality, but also efficiently handles large-scale networks.

#### Definition 8

In undirected networks, modularity is commonly used to measure the effectiveness of community division. The value of modularity typically ranges from 0.5 to 1^43^, and it is defined as:

6 $$Q=\frac{1}{{2 \times \kappa }} \times \sum\limits_{{e,h}} {\left[ {{E_{eh}} - \frac{{{\alpha _e} \times {\alpha _h}}}{{2 \times \kappa }}} \right]} \times \delta \left( {{G_e},{G_h}} \right)$$

where *κ* represents the number of edges in the network. If an edge exists between nodes ${v_e}$ and ${v_h}$, ${E_{eh}}=1$; otherwise, ${E_{eh}}=0$. ${\alpha _e}$ and ${\alpha _h}$ indicate the degrees of nodes ${v_e}$ and ${v_h}$, respectively. ${G_e}$ and ${G_h}$ denote the communities of nodes ${v_e}$ and ${v_h}$. If nodes ${v_e}$ and ${v_h}$ belong to the same community, $\delta \left( {{G_e},{G_h}} \right)=1$; otherwise, $\delta \left( {{G_e},{G_h}} \right)=0$.

#### **Definition 9**^44^

In undirected networks, the gain of modularity is given by:

7 $$\Delta Q=\frac{{\alpha _{e}^{G}}}{{2 \times \kappa }} - \frac{{\sum\limits_{{tot}}^{G} {{\alpha _e}} }}{{2 \times {\kappa ^2}}}$$

where $\alpha _{e}^{G}$ represents the degree of node ${v_e}$ in community *G*, and $\sum\limits_{{tot}}^{G} {}$indicates the number of edges connected to community *G*.

### Minimum adjustment consensus model

The minimal adjustment consensus model (MACM) seeks to reduce the difference between the initial preferences of the DMs and their modified preferences. Dong et al.^45^ were the first to propose the specific optimization model for the MACM, as follows:

8 $$\begin{gathered} \boldsymbol{M}1\quad \hbox{min} \;\sum\limits_{{e=1}}^{k} {d^{\prime}\left( {{{\bar {o}}_e},{o_e}} \right)} \hfill \\ s.t.\left\{ \begin{gathered} \bar {o}={\mathbb{F}}\left( {{{\bar {o}}_1},{{\bar {o}}_2}, \ldots ,{{\bar {o}}_k}} \right)\quad \left( {8.1} \right) \hfill \\ \left| {\bar {o} - {{\bar {o}}_e}} \right| \leqslant \varepsilon \quad \quad \quad \quad \;\;\left( {8.2} \right) \hfill \\ \end{gathered} \right. \hfill \\ \end{gathered}$$

where ${o_e}$ and ${\bar {o}_e}$ represent the initial and adjusted preferences of DM ${v_e}$, respectively. $d^{\prime}\left( {{{\bar {o}}_e},{o_e}} \right)$ indicates the deviation distance before and after the preference adjustment. $\bar {o}$ represents the collective preference of all DMs. ${\mathbb{F}}$ denotes the aggregation operator, and $\varepsilon$ represents the acceptable deviation threshold.

### Minimum cost consensus model

The minimal cost consensus model (MCCM) focuses on minimizing the cost of the DMs’ to adjust their opinions, ensuring that they achieve the most economic consensus. Zhang et al.^46^ developed a threshold-based MCCM, as follows:

9 $$\begin{gathered} {M}2\,\,\,\,\,\,\,\,\,\min \,\,\sum\limits_{{e = 1}}^{k} {c_{e} \times } \left| {\bar{o}_{e} - o_{e} } \right| \hfill \\ s.t.\left\{ \begin{gathered} \bar{o} = \mathbb{F}\left( {\bar{o}_{1}{,}\bar{o}_{2} {,} \ldots {,}\bar{o}_{k} } \right)\,\,\,\,\,\,\,\,\,\left( {9.1} \right) \hfill \\ CD\left( {\bar{o}_{e} {,}\bar{o}} \right) \ge \eta \,\,\,\,\,\,\,\,\,\,\,\,\,\,\,\,\,\,\,\,\,\,\left( {9.2} \right) \hfill \\ \end{gathered} \right. \hfill \\ \end{gathered}$$

where ${c_e}$ represents the unit adjustment cost, and *η* represents the consensus threshold.

## A CRP for LGDM in dynamic trust networks

### Problem description

Let the set of DMs be ${\boldsymbol{V}}=\left\{ {{v_e}|e=1,2,...,k} \right\}$. Let ${\boldsymbol{X}}=\left\{ {{x_i}|i=1,2,...,m} \right\}$ and ${\boldsymbol{Y}}=\left\{ {{y_j}|j=1,2, \ldots ,n} \right\}$ represent the set of alternative solutions and attributes, respectively. Suppose there are $k \geqslant 20$ DMs, *m* alternatives, and *n* attributes are considered. The preferences of DMs toward the alternative solutions are expressed through uncertain linguistic variables. The evaluation matrix of the alternatives provided by DM ${v_e}$ is denoted as ${{\boldsymbol{P}}_e}={\left( {\tilde {p}_{{ij}}^{e}} \right)_{m \times n}}$, where uncertain linguistic variable $\tilde {p}_{{ij}}^{e}$ represents the preference information of DM ${v_e}$ for the alternative ${x_i}$ regarding attribute ${y_j}$. This paper defines the problem as the process of ranking a set of alternatives based on their attributes in LGDM, with the goal of selecting the optimal alternative. For clarity, the abbreviations used in this paper are summarized in Table 3, and the definitions of the main symbols are provided in Table 4.

**Table 3:** Abbreviations used in this paper.

Abbreviations	Full term
CRP	Consensus reaching process
DI	Dominance index
DM	Decision-maker
DMs	Decision-makers
DMP	Decision-making processes
GCL	Group consensus level
GDM	Group decision-making
HTN	Hybrid trust network
LGDM	Large-scale group decision-making
MACM	Minimal adjustment consensus model
MCCM	Minimal cost consensus model
MCMFMCL-MOOCM	A MOOCM driven by minimum cost, maximum fairness and maximizing consensus level
MCMF-MO-LGDM	MOOCM for LGDM targeting minimum cost and maximum fairness
MOOCM	Multi-objective optimization consensus model
MSCM	Maximum satisfaction consensus model
PV	Photovoltaic
SNA	Social network analysis

**Table 4:** Definitions of symbols used in this paper.

Symbols	Definition	Symbols	Definition
***V***	The set of DMs	${v_e}$	The *e*-th DM
*k*	The number of DMs	***X***	The set of alternatives
${x_i}$	The *i*-th alternative	*m*	The number of alternatives
***Y***	The set of attributes	${y_j}$	The *j*-th attribute
*n*	The number of attributes	${{\boldsymbol{P}}_e}$	The evaluation matrix provided by DM ${v_e}$
$\tilde {p}_{{ij}}^{e}$	The uncertain linguistic variable expressing DM ${v_e}$’s preference for alternative ${x_i}$ on attribute ${y_j}$	${\boldsymbol{S}}$	Linguistic term set
$T{S_{eh}}$	The trust score of ${v_e}$ to ${v_h}$	*Q*	The modularity
$\Delta Q$	The gain of modularity	${o_e}$	The initial preferences of DM ${v_e}$
${\bar {o}_e}$	The adjusted preferences of DM ${v_e}$	$\bar {o}$	The collective preference of all DMs
${c_e}$	The DM ${v_e}$’s unit adjustment cost	$SI{M_{eh}}$	The preference similarity between DM ${v_e}$ and ${v_h}$
$T{D_{eh}}$	The trust-similarity score between DM ${v_e}$ and ${v_h}$	$HT{D_{eh}}$	The undirected trust-similarity score between DM ${v_e}$ and ${v_h}$
$TS_{{eh}}^{{\left( {r+1} \right)}}$	The trust score between DMs ${v_e}$ and ${v_h}$ in the (*r* + 1)*-*th iteration	$w_{h}^{{\left( r \right)}}$	The DM ${v_h}$’ weight in the *r-*th iteration
${G_l}$	The *l*-th subgroup obtained from clustering	$w_{l}^{{\left( r \right)}}$	The Subgroup ${G_l}$’ weight in the *r-*th iteration
$w_{{e,l}}^{{\left( r \right)}}$	The relative weight of DM ${v_e}$ within subgroup ${G_l}$ in the *r*-th iteration	$ICD_{{e,l}}^{{\left( r \right)}}$	The consensus level of DM ${v_e}$ in subgroup ${G_l}$ during the *r-*th iteration
${\boldsymbol{P}}_{l}^{{\left( r \right)}}$	The evaluation matrix in subgroup ${G_l}$ at the *r-*th iteration	${\boldsymbol{P}}_{G}^{{\left( r \right)}}$	The group preference matrix at the *r*-th iteration
$CL_{l}^{{\left( r \right)}}$	The consensus level of subgroup ${G_l}$ at the *r-*th iteration	$r{v_e}$	The payoff value obtained by DM ${v_e}$
$r{v_h}$	The payoff value obtained by DM ${v_h}$	$\overline {{rv}}$	The average payoff value of all DMs
${\bar {{\boldsymbol{P}}}_l}$	The modified subgroup ${G_l}$’s preference matrix	$\overline {{GCL}}$	The adjusted GCL
*η*	Consensus threshold	*β*	Weight parameter in HTN
*ω*	Attribute weights	${\sigma _L}$	Lower bound of similarity threshold in dynamic trust updating
${\sigma _U}$	Upper bound of similarity threshold in dynamic trust updating	*r*	Iteration index in the CRP
*z*	The number of subgroups	${g_l}$	Number of DMs in subgroup ${G_l}$
${\mathbb{F}}$	The aggregation operator	${\gamma _e}$	Adjustment parameter of DM ${v_e}$

### Trust network updating process

Most studies on trust networks in LGDM assume that trust relationships are static, and only a few consider their dynamic evolution. Additionally, methods for updating trust still need to be improved. In order to more effectively depict the evolution of trust between DMs, this paper proposes a dynamic mechanism for updating the trust network.

#### Construction of hybrid trust network

Trust plays an important role in the CRP, as higher trust leads to faster consensus^47^ and provides an effective foundation for dimensionality reduction in LGDM^48^. In social network LGDM, DMs are linked through complex trust relationships. Previous research methods indicate that trust relationships consist of two aspects. On the one hand, DMs are already acquainted prior to the DMP and establish initial trust based on subjective evaluations. On the other hand, trust relationships are influenced by the interaction of opinions during the DMP. Initial trust relationships are built based on DMs’ experiences, their authority in relevant fields, and subjective evaluations from past interactions^28^. Therefore, DMs are more likely to trust other decision-makers with similar views and establish trust relationships with them^49,50^. It is assumed that a greater similarity in opinions between DMs leads to a higher trust level, while a lower opinion similarity results in a lower trust level. Therefore, this paper integrates initial trust relationships with opinion similarity to build an initial HTN.

*Step 1* DMs first assess their trust in other DMs and then evaluate the alternatives. Trust evaluations are typically obtained in the form of fuzzy sets^51^, real numbers^52^, or others^53^. The facilitator collects and processes the evaluation information to establish the initial trust matrix. Let ${\boldsymbol{T}}{\boldsymbol{M}}={\left( {T{S_{eh}}} \right)_{k \times k}}$ represent the initial trust matrix constructed from the initial trust relationships among DMs:

$${\boldsymbol{T}}{\boldsymbol{M}}={\left[ {\begin{array}{*{20}{c}} 0&{T{S_{12}}}& \ldots &{T{S_{1k}}} \\ {T{S_{21}}}&0& \ldots &{T{S_{2k}}} \\ \vdots & \vdots & \ddots & \vdots \\ {T{S_{k1}}}&{T{S_{k2}}}& \ldots &0 \end{array}} \right]_{k \times k}}$$

*Step 2* During the DMP, trust relationships among DMs constantly change along with the interaction of opinions and are affected by the similarity of opinions. Thus, integrating trust relationships with opinion similarity to create an HTN allows for a more comprehensive reflection of dynamic trust relationships. The opinion similarity among DMs can be measured by calculating the distance of the opinion evaluation matrix for alternatives. The opinion similarity between any two DMs is given by^54^ :

10 $$SI{M_{eh}}=1 - d^{\prime}\left( {{{\boldsymbol{P}}_e} - {{\boldsymbol{P}}_h}} \right)=1 - \frac{1}{{m \times n}} \times \sum\limits_{{i=1}}^{m} {\sum\limits_{{j=1}}^{n} {\left| {\tilde {p}_{{ij}}^{e} - \tilde {p}_{{ij}}^{h}} \right|} }$$

*Step 3* Based on the above analysis, the trust-similarity score between DM ${v_e}$ and ${v_h}$ is calculated as:

11 $$T{D_{eh}}=\beta \times T{S_{eh}}+\left( {1 - \beta } \right) \times SI{M_{eh}}$$

where $\beta \in \left[ {0,1} \right]$ represents the weight parameter.

*Step 4*^48^ Since the values of $T{S_{he}}$ and $T{S_{eh}}$ are not necessarily identical, it is required to adjust the directed social matrix ${\boldsymbol{T}}{\boldsymbol{M}}={\left( {T{S_{eh}}} \right)_{k \times k}}$ for an undirected social matrix ${\boldsymbol{A}}{\boldsymbol{T}}{\boldsymbol{M}}={\left( {AT{S_{eh}}} \right)_{k \times k}}$.

12 $$AT{S_{eh}}=\hbox{min} \left\{ {T{S_{eh}},T{S_{he}}} \right\}$$

Thus, we obtain $AT{S_{eh}}=AT{S_{he}}$, and the undirected trust-similarity score between DM ${v_e}$ and ${v_h}$ is:

13 $$HT{D_{eh}}=\beta \times AT{S_{eh}}+\left( {1 - \beta } \right) \times SI{M_{eh}}$$

Finally, by using Eq. (13), the HTN matrix ${\boldsymbol{H}}{\boldsymbol{T}}{\boldsymbol{M}}={\left( {HT{D_{eh}}} \right)_{k \times k}}$ can be obtained, where $HT{D_{eh}} \in \left[ {0,1} \right]$. This matrix more accurately reflects the impact of the original social connections and the opinion similarity on trust relationships.

#### Trust network updating

Most existing studies assume that trust relationships are static during the DMP, but some studies focus on the evolution of trust. In fact, as DMs interact, preferences, trust relationships and social influence will change^55^. Unlike static trust network, dynamic trust network can be adjusted based on interactions, changes in opinions, and the evolution of the DMP. Therefore, this paper proposes a dynamic trust network updating mechanism.

This paper comprehensively considers the combined effect of trust relationships and preference similarity on trust network updates and puts forward the following two hypotheses: (1) Trust relationship among DMs is shaped by preference similarity; (2) DMs tend to place greater trust in other decision-makers with higher preference similarity. Through a feedback mechanism, the preferences of DMs will be continually adjusted, thereby causing changes in preference similarity and promoting the dynamic update of the HTN matrix.

On this basis, this study derives updated preference similarity from the revised preference matrix. If the new preference similarity exceeds the upper limit of the similarity threshold, the trust score between DMs increases. If the new preference similarity is between the upper and lower limits of the threshold, the trust score decreases. If it falls below the lower limit, the minimum level of trust, i.e., the original trust score, is retained. Therefore, the dynamic trust network update rule is as follows:

14 $$TS_{{eh}}^{{\left( {r+1} \right)}}=\left\{ \begin{gathered} TS_{{eh}}^{{\left( r \right)}}+\left( {1 - TS_{{eh}}^{{\left( r \right)}}} \right) \times \frac{{SIM_{{eh}}^{{\left( r \right)}} - {\upsigma _U}}}{{1 - {\upsigma _U}}},\;SIM_{{eh}}^{{\left( r \right)}} \in \left( {{\upsigma _U},1} \right] \hfill \\ TS_{{eh}}^{{\left( r \right)}} - \left( {1 - TS_{{eh}}^{{\left( r \right)}}} \right) \times \frac{{{\upsigma _U} - SIM_{{eh}}^{{\left( r \right)}}}}{{{\upsigma _U} - {\upsigma _L}}},\;SIM_{{eh}}^{{\left( r \right)}} \in \left( {{\upsigma _L},{\upsigma _U}} \right] \hfill \\ TS_{{eh}}^{{\left( r \right)}},\quad \quad \quad \quad \quad \quad \quad \quad \quad \quad \;\,SIM_{{eh}}^{{\left( r \right)}} \in \left[ {0,{\upsigma _L}} \right) \hfill \\ \end{gathered} \right.$$

15 $$TS_{{he}}^{{\left( {r+1} \right)}}=\left\{ \begin{gathered} TS_{{he}}^{{\left( r \right)}}+\left( {1 - TS_{{he}}^{{\left( r \right)}}} \right) \times \frac{{SIM_{{he}}^{{\left( r \right)}} - {\upsigma _U}}}{{1 - {\upsigma _L}}},\;SIM_{{he}}^{{\left( r \right)}} \in \left( {{\upsigma _U},1} \right] \hfill \\ TS_{{he}}^{{\left( r \right)}} - \left( {1 - TS_{{he}}^{{\left( r \right)}}} \right) \times \frac{{{\upsigma _U} - SIM_{{he}}^{{\left( r \right)}}}}{{{\upsigma _U} - {\upsigma _L}}},\;SIM_{{he}}^{{\left( r \right)}} \in \left( {{\upsigma _L},{\upsigma _U}} \right] \hfill \\ TS_{{he}}^{{\left( r \right)}},\quad \quad \quad \quad \quad \quad \quad \quad \quad \quad \;\,SIM_{{he}}^{{\left( r \right)}} \in \left[ {0,{\upsigma _L}} \right) \hfill \\ \end{gathered} \right.$$

where $TS_{{eh}}^{{\left( {r+1} \right)}}$ represents the trust score between DMs ${v_e}$ and ${v_h}$ in the (*r* + 1)*-*th round, and ${\upsigma _L}$, ${\upsigma _U}$ respectively denote the lower and upper limits of the similarity threshold. They are used to distinguish the trust updating intervals under different similarity levels, and they can be set by DMs or determined based on experience or historical data.

After the trust network is updated, the hybrid trust degree is updated to $$HTD_{{eh}}^{{\left( {r+1} \right)}}=\beta \times ATS_{{eh}}^{{\left( {r+1} \right)}}+\left( {1 - \beta } \right)$$$$\times SIM_{{eh}}^{{\left( {r+1} \right)}}$$ in combination with preference similarity. The updated HTN matrix is $${\boldsymbol{H}}{\boldsymbol{T}}{{\boldsymbol{M}}^{\left( {r+1} \right)}}={\left( {HTD_{{eh}}^{{\left( {r+1} \right)}}} \right)_{k \times k}}$$. The update of dynamic trust network will provide a foundation for further clustering of the DMs.

#### Dynamic weights based on trust and similarity

In a social trust network, decision makers with a higher trust level tend to exert greater influence in GDM compared to those with a lower trust level^28,56^. Therefore, the DM with the highest trust level should be assigned the maximum weight. It is crucial to aggregate individual preferences based on the DMs’ weights to obtain group preferences, as this process may further affect the GCL. Hence, to achieve effective consensus, it is vital to reasonably determine the DMs’ weights. This paper uses hybrid trust as the basis for determining the weight of each DM. Thus, the DM ${v_h}$’ weight in the *r-*th ($r \in \left\{ {0,1, \ldots ,R} \right\}$) iteration is:

16 $$w_{h}^{{\left( r \right)}}=\frac{{\sum\limits_{{e=1,e \ne h}}^{k} {HT{D_{eh}}} }}{{\sum\limits_{{h=1}}^{k} {\sum\limits_{{e=1,e \ne h}}^{k} {HT{D_{eh}}} } }}$$

where $w_{h}^{{\left( r \right)}} \in \left[ {0,1} \right]$, and satisfies $\sum\limits_{{h=1}}^{k} {w_{h}^{{\left( r \right)}}} =1$. Each subgroup’s weight is determined by the weights of the DMs within it and can be expressed as:

17 $$w_{l}^{{\left( r \right)}}=\sum\limits_{{{v_h} \in {G_l}}} {w_{h}^{{\left( r \right)}}}$$

where $w_{l}^{{\left( r \right)}} \in \left[ {0,1} \right]$, and satisfies $\sum\limits_{{l=1}}^{z} {w_{l}^{{\left( r \right)}}} =1$. Subgroup ${G_l}\left( {l=1,2, \ldots ,z} \right)$’s weight is closely related to the number and weight of DMs in the subgroup.

If the aggregation of preferences within the subgroups is required, the weight of each DM in the subgroup must be specified. Assuming DM ${v_e}$ belongs to subgroup ${G_l}$, the DM ${v_e}$’ weight in the subgroup is:

18 $$w_{{e,l}}^{{\left( r \right)}}=\frac{{w_{e}^{{\left( r \right)}}}}{{w_{l}^{{\left( r \right)}}}}$$

where $w_{{e,l}}^{{\left( r \right)}} \in \left[ {0,1} \right]$ and satisfies $\sum\limits_{{e=1}}^{{{g^l}}} {w_{{e,l}}^{{\left( r \right)}}=1}$. ${g^l}$ indicates the number of DMs in subgroup ${G_l}$.

### CRP based on a multi-objective optimization model

#### Consensus measurement

Typically, the CRP proceeds in two phases, namely consensus measurement and feedback adjustment^57^. This process works to reduce differences of opinion among DMs and reach a decision result that most members can accept by adjusting their preferences^50^. Another goal is to maximize GCL. Consensus measurement assesses the current level of agreement and determines a preset threshold is met. It is the basis of feedback and by calculating the consensus level of DMs, it reveals how much agreement or disagreement exists among them. This supports a targeted feedback process, which enhances acceptance toward the final decision result^58^. Feedback adjustment provides suggestions for DMs to adjust their opinions, thereby further increasing the consensus level. Hence, this study assesses the consensus level based on the similarity of opinions and conducts calculations from three dimensions: individual, subgroup, and overall GCL.

##### Definition 10

The individual consensus level refers to the proximity between the evaluation matrix of a single DM’s and the evaluation matrix of the subgroup formed by weighted aggregation. The consensus level of DM ${v_e}$ in subgroup ${G_l}$ during the *r-*th iteration is defined as:

19 $$ICD_{{e,l}}^{{\left( r \right)}}=1 - d^{\prime}\left( {{\boldsymbol{P}}_{e}^{{\left( r \right)}} - {\boldsymbol{P}}_{l}^{{\left( r \right)}}} \right)=1 - \frac{1}{{m \times n}} \times \sum\limits_{{i=1}}^{m} {\sum\limits_{{j=1}}^{n} {\left| {\tilde {p}_{{ij}}^{{e,\left( r \right)}} - \tilde {p}_{{ij}}^{{l,\left( r \right)}}} \right|} }$$

where ${\boldsymbol{P}}_{l}^{{\left( r \right)}}$ is taken as the evaluation matrix in subgroup ${G_l}$ at the *r-*th iteration:

20 $${\boldsymbol{P}}_{l}^{{\left( r \right)}}={\left( {\tilde {p}_{{ij}}^{{l,\left( r \right)}}} \right)_{m \times n}}=\sum\limits_{{e=1}}^{{{g^l}}} {\sum\limits_{{i=1}}^{m} {\sum\limits_{{j=1}}^{n} {w_{{e,l}}^{{\left( r \right)}} \times \tilde {p}_{{ij}}^{{e,\left( r \right)}}} } }$$

Thus, based on the weight of each subgroup and formula (20), the group preference matrix at the *r-*th iteration is obtained as:

21 $${\boldsymbol{P}}_{G}^{{\left( r \right)}}={\left( {\tilde {p}_{{ij}}^{{G,\left( r \right)}}} \right)_{m \times n}}=\sum\limits_{{l=1}}^{z} {\sum\limits_{{i=1}}^{m} {\sum\limits_{{j=1}}^{n} {w_{l}^{{\left( r \right)}} \times \tilde {p}_{{ij}}^{{l,\left( r \right)}}} } }$$

##### Definition 11

The subgroup consensus level can be calculated by taking a weighted average of the individual consensus levels. The consensus level of subgroup ${G_l}$ at the *r-*th iteration is defined as:

22 $$CL_{l}^{{\left( r \right)}}=\sum\limits_{{e=1}}^{{{g^l}}} {w_{{e,l}}^{{\left( r \right)}} \times ICD_{{e,l}}^{{\left( r \right)}}}$$

##### Definition 12

The GCL at the *r-*th iteration is defined as:

$$GC{L^{\left( r \right)}}=\sum\limits_{{l=1}}^{z} {w_{l}^{{\left( r \right)}} \times {\kern 1pt} {\kern 1pt} } CL_{l}^{{\left( r \right)}}=\sum\limits_{{l=1}}^{z} {w_{l}^{{\left( r \right)}}} \times \sum\limits_{{e=1}}^{{{g^l}}} {w_{{e,l}}^{{\left( r \right)}} \times ICD_{{e,l}}^{{\left( r \right)}}} =$$

23 $$\sum\limits_{{l=1}}^{z} {w_{l}^{{\left( r \right)}}} \times \sum\limits_{{e=1}}^{{{g^l}}} {\frac{{w_{e}^{{\left( r \right)}}}}{{w_{l}^{{\left( r \right)}}}} \times ICD_{{e,l}}^{{\left( r \right)}}} =\sum\limits_{{l=1}}^{z} {\sum\limits_{{e=1}}^{{{g^l}}} {w_{e}^{{\left( r \right)}} \times ICD_{{e,l}}^{{\left( r \right)}}} }$$

where $$GC{L^{\left( r \right)}} \in \left[ {0,1} \right]$$.

When $GC{L^{\left( r \right)}}=1$, it indicates that all DMs in the group are in complete agreement. However, in actual DMP, such a situation is almost impossible. Previous research often adopts the concept of “soft consensus” to describe the consensus level attained among DMs^13^. Typically, a threshold *η* is set to determine whether the group consensus has reached a level acceptable to most DMs. If and only if $GC{L^{\left( r \right)}} \geqslant \eta$, it means that the group has reached a consensus and can proceed with alternative ranking and selection. Otherwise, a feedback mechanism should be employed to modify DMs’ opinion to enhance the consensus level until the threshold is achieved or the maximum iterations are completed.

#### Clustering process

The HTN embodies both trust relationships and preference similarity among DMs, which facilitates efficient clustering. Since this network is essentially an undirected weighted graph, this paper employs the Louvain algorithm to divide its subgroups. Dividing the HTN into subgroups is conducive to reducing the complexity of LGDM. Unlike the conventional Louvain algorithm, the proposed method integrates both preference similarity and trust relations, making it more comprehensive than prior approaches. This method enhances cohesion within the subgroup as well as clustering quality, laying a stronger basis for the subsequent CRP. Algorithm 1 illustrates the Louvain algorithm steps.

**Algorithm 1:**
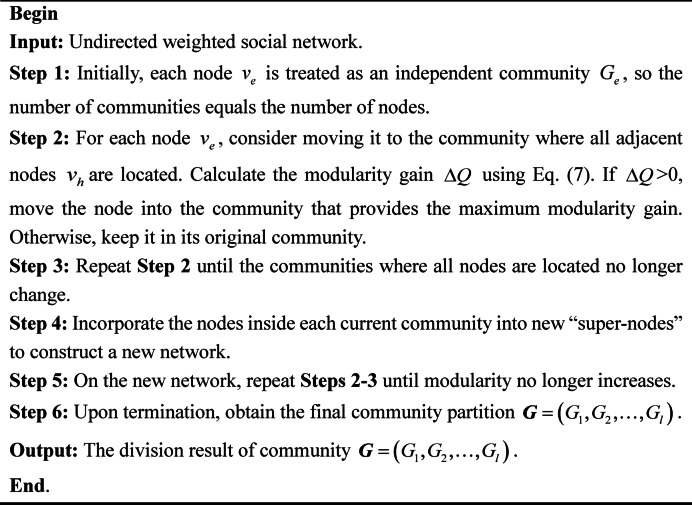
Louvain algorithm based on HTN in undirected weighted network.

#### Multi-objective optimization consensus model

To achieve consensus effectively, multiple objectives are usually considered simultaneously, including adjustment cost, fairness and the consensus level. It is important to reflect the preferences of as many DMs as possible. This helps to avoid overlooking the opinions of some participants during the DMP. Therefore, this paper proposes a MOOCM driven by minimum cost, maximum fairness and maximization of consensus level.

##### Definition 13

In GDM, fairness is an important condition that affects whether the final consensus result can be widely accepted. If the group preference leans too much toward a few authoritative members while ignoring the opinions of the majority of regular members, even if a formal consensus is reached, it may still trigger dissatisfaction and resistance among some members. Therefore, the fairness degree of preference adjustment is defined as^59^:

24 $$FD=1 - \frac{{\sum\limits_{{e=1}}^{k} {\sum\limits_{{h=1}}^{k} {\left| {r{v_e} - r{v_h}} \right|} } }}{{2 \times {k^2} \times \overline {{rv}} }}$$

where $r{v_e}$ and $r{v_h}$represent the payoff values obtained by different DM ${v_e}$ and ${v_h}$. $\overline {{rv}}$ indicates the average payoff value of all DMs. The payoff value of DM ${v_e}$ is given by: $r{v_e}={c_e} \times \left| {{o_e} - {{\bar {o}}_e}} \right|$.

While minimizing cost and maximizing fairness, the model also aims to maximize the consensus level. These multi-goals not only emphasize economic efficiency, but also take into account social equity and practical feasibility, making the optimized results more reasonable and acceptable in practice. Based on the above, and combined the study by Labella et al.^60^, a MOOCM driven by minimum cost, maximum fairness and maximizing consensus level is (MCMFMCL-MOOCM) formulated as follows:

25 $$\begin{gathered} \mathrm{M}3\quad \hbox{min} \;{f_1}\;=\sum\limits_{{e=1}}^{k} {{c_e} \times \left| {{{\bar {o}}_e} - {o_e}} \right|} \hfill \\ \quad \quad \;\hbox{max} \;{f_2}=\;1 - \frac{1}{{2 \times {k^2} \times \overline {{rv}} }} \times \sum\limits_{{e=1}}^{k} {\sum\limits_{{h=1}}^{k} {\left| {r{v_e} - r{v_h}} \right|} } \hfill \\ \quad \quad \;\hbox{max} \;{f_3}\;=1 - \sum\limits_{{l=1}}^{z} {\sum\limits_{{e=1}}^{{{g^l}}} {{w_e} \times d\prime \left| {{{\bar {o}}_e} - {{\bar {{\boldsymbol{P}}}}_l}} \right|} } \hfill \\ s.t.\left\{ \begin{gathered} {{\bar {o}}_e}={\gamma _e} \times {{\boldsymbol{P}}_l}+\left( {1 - {\gamma _e}} \right) \times {o_e}\quad \quad \quad \quad \quad \;\;\,\left( {25.1} \right) \hfill \\ {{\boldsymbol{P}}_l}=\sum\limits_{{e=1}}^{{{g^l}}} {{w_{e,l}} \times {o_e},\;l=1,2, \ldots ,z\quad \quad \quad \quad \;\left( {25.2} \right)} \hfill \\ {{\bar {{\boldsymbol{P}}}}_l}=\sum\limits_{{e=1}}^{{{g^l}}} {{w_{e,l}} \times {{\bar {o}}_e},\;l=1,2, \ldots ,z\quad \quad \quad \quad \;\left( {25.3} \right)} \hfill \\ \overline {{GCL}} =1 - \sum\limits_{{l=1}}^{z} {\sum\limits_{{e=1}}^{{{g^l}}} {{w_e} \times d\prime \left| {{{\bar {o}}_e} - {{\bar {{\boldsymbol{P}}}}_l}} \right| \geqslant \eta \quad \,\,\,\,\left( {25.4} \right)} } \hfill \\ r{v_e}={c_e} \times \left| {{o_e} - {{\bar {o}}_e}} \right|,\;e=1,2, \ldots ,k\quad \quad \quad \;\,\left( {25.5} \right) \hfill \\ \overline {{rv}} =\frac{1}{k}\sum\limits_{{e=1}}^{k} {r{v_e}\quad \quad \quad \quad \quad \quad \quad \quad \quad \quad \;\,\left( {25.6} \right)} \hfill \\ 0 \leqslant {\gamma _e} \leqslant 1,\;e=1,2, \ldots ,k\quad \quad \quad \quad \quad \quad \;\;\,\left( {25.7} \right) \hfill \\ \end{gathered} \right. \hfill \\ \end{gathered}$$

where ${o_e}$ and ${\bar {o}_e}$ denote the original and revised preferences for DM ${v_e}$. ${{\boldsymbol{P}}_l}$ and ${\bar {{\boldsymbol{P}}}_l}$ indicate the initial and modified preferences within subgroup ${G_l}$. $\overline {{GCL}}$ reflects the adjusted GCL.

In Model M3, Objective Function 1 aims to minimize the cost of opinion adjustment. Objective Function 2 seeks to improve the fairness of the payoff. Objective Function 3 is dedicated to maximizing the consensus level. Constraints (25.2) and (25.3) are used to calculate the initial and adjusted preferences of the subgroups. Constraint (25.4) ensures that the consensus threshold is met. Constraints (25.1) and (25.7) define the adjustment parameters. Constraints (25.5) and (25.6) are applied to compute the utility.

At present, a range of techniques are employed to address multi-objective optimization models, among them genetic algorithms, particle swarm optimization, and simulated annealing. These methods can obtain approximate optimal solutions to some extent. However, when dealing with complex constraints, conflicting objectives and large solution spaces, they often have limitations, including slow convergence speed, uneven distribution of solutions or easily trapped into local optima. In comparison, the NSGA-II algorithm shows more prominent advantages for multi-objective optimization. It ensures solution convergence through the elite preservation strategy of non-dominated sorting and maintains the diversity of the solution set by introducing crowding distance. Moreover, it adapts well to LGDM optimization problems with relatively low computational complexity. In particular, when handling nonlinear constraints, this algorithm effectively prevents entrapment in local optima^61^.

Hence, this paper employs NSGA-II to solve MOOCM proposed previously. The main steps are shown in Algorithm 2. This ensures that a well distributed and diverse Pareto-optimal solution set can be achieved while balancing three aims, which are cost minimization, fairness maximization, and consensus improvement. The solution generated by this method not only provides multiple feasible options for subsequent CRP but also offers DMs a basis for choice under different trade-offs. Based on these solutions, the preference matrices of DMs are adjusted, and the trust network is updated, further strengthening the scientific validity and logical consistency within the DMP.

**Algorithm 2:**
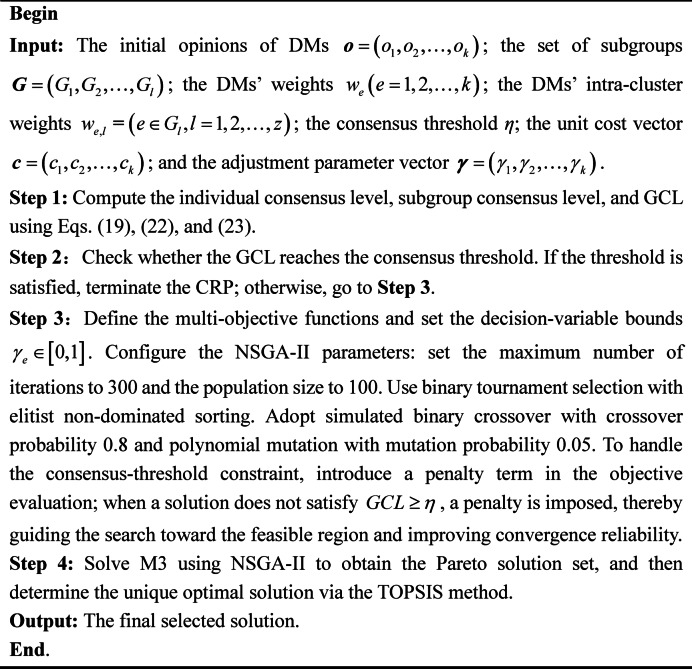
Solving the MOOCM based on NSGA-II.

### Decision-making process

**Fig. 1:**
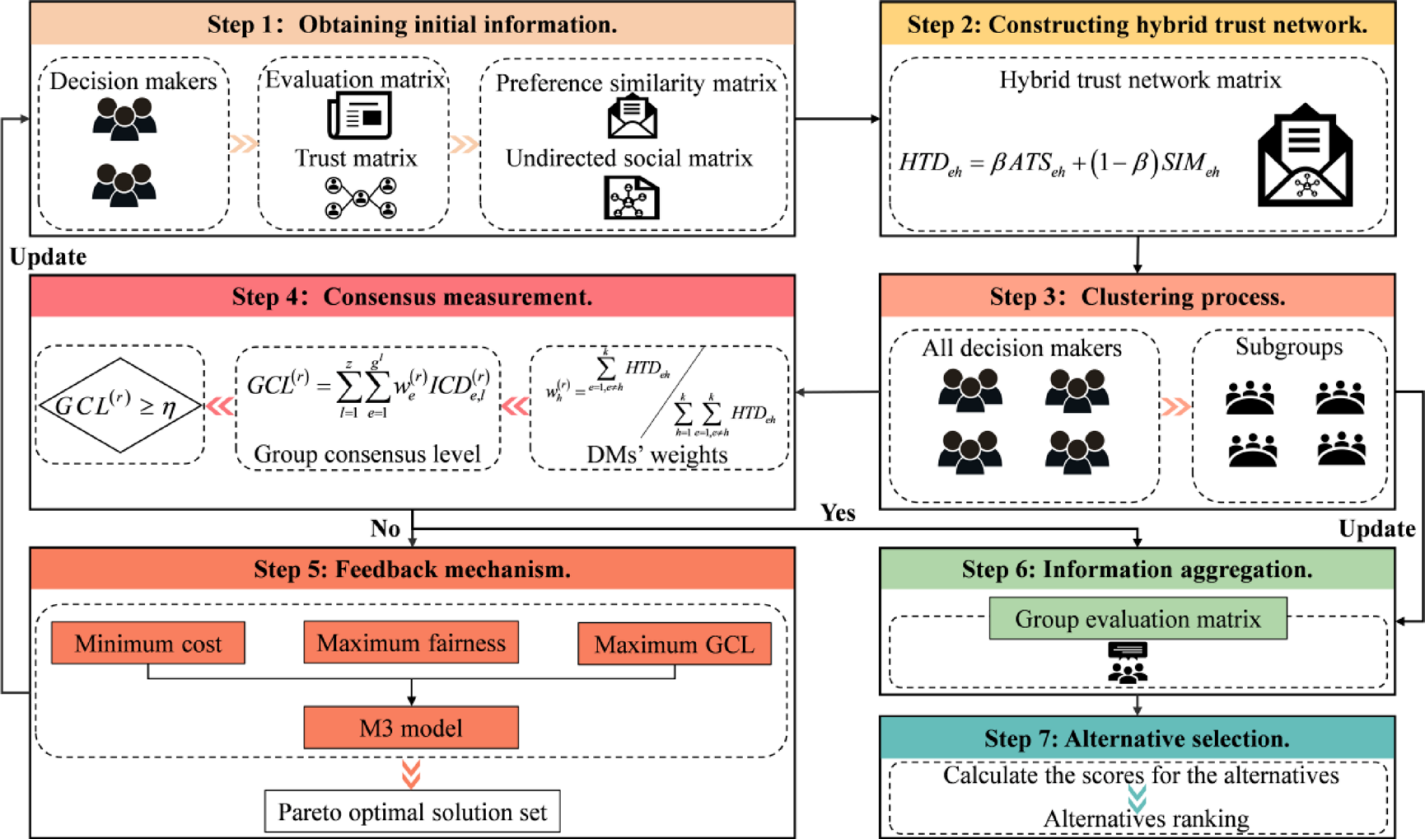
Decision flowchart of the MOOCM based on dynamic trust networks.

From the preceding analysis, a MOOCM employing dynamic trust networks is proposed for LGDM. The decision process is illustrated in Fig. 1, with the core steps outlined as follows.

*Step 1* Obtain initial information. First, acquire the directed social matrix among DMs ${\boldsymbol{T}}{{\boldsymbol{M}}^{\left( r \right)}}={\left( {TS_{{eh}}^{{\left( r \right)}}} \right)_{k \times k}}$ and the preference information matrix ${\boldsymbol{P}}_{e}^{{\left( r \right)}}={\left( {\tilde {p}_{{ij}}^{{e,\left( r \right)}}} \right)_{m \times n}}$. Then, calculate the preference similarity among DMs using Eq. (10) to obtain the similarity matrix ${\boldsymbol{S}}{{\boldsymbol{M}}^{\left( r \right)}}={\left( {SIM_{{eh}}^{{\left( r \right)}}} \right)_{k \times k}}$. Finally, adjust the directed social matrix using Eq. (12) to derive the undirected social matrix ${\boldsymbol{A}}{\boldsymbol{T}}{{\boldsymbol{M}}^{\left( r \right)}}={\left( {ATS_{{eh}}^{{\left( r \right)}}} \right)_{k \times k}}$.

*Step 2* HTN construction. Combine the undirected social matrix $${\boldsymbol{A}}{\boldsymbol{T}}{{\boldsymbol{M}}^{\left( r \right)}}=$$$${\left( {ATS_{{eh}}^{{\left( r \right)}}} \right)_{k \times k}}$$ and the similarity matrix $${\boldsymbol{S}}{{\boldsymbol{M}}^{\left( r \right)}}={\left( {SIM_{{eh}}^{{\left( r \right)}}} \right)_{k \times k}}$$ using Eq. (13) to form the HTN matrix $${\boldsymbol{H}}{\boldsymbol{T}}{{\boldsymbol{M}}^{\left( r \right)}}={\left( {HTD_{{eh}}^{{\left( r \right)}}} \right)_{k \times k}}$$.

*Step 3* Clustering process. Based on the HTN matrix $${\boldsymbol{H}}{\boldsymbol{T}}{{\boldsymbol{M}}^{\left( r \right)}}={\left( {HTD_{{eh}}^{{\left( r \right)}}} \right)_{k \times k}}$$, perform clustering according to Algorithm 1. Obtain the clustering result $${\boldsymbol{G}}=$$$$\left( {{G_1},{G_2}, \ldots ,{G_l}} \right)$$.

*Step 4* Consensus measurement. First, use Eqs. (16)–(18) to compute individual DM weights, subgroup weights, and relative weights assigned to DMs in each subgroup. Next, combine DMs’ preference information through Eqs. (20) and (21). This gives the subgroup preference matrix ${\boldsymbol{P}}_{l}^{{\left( r \right)}}={\left( {\tilde {p}_{{ij}}^{{l,\left( r \right)}}} \right)_{m \times n}}$ and the group preference matrix ${\boldsymbol{P}}_{G}^{{\left( r \right)}}={\left( {\tilde {p}_{{ij}}^{{G,\left( r \right)}}} \right)_{m \times n}}$. Subsequently, Eqs. (19), (22), and (23) are applied to evaluate consensus at three levels: individual DM, subgroup, and the group as a whole. If $GC{L^{\left( r \right)}} \geqslant \eta$, consensus is considered reached and the procedure advances to Step 6; otherwise, proceed to Step 5.

*Step 5* Feedback mechanism. By solving Model M3, the Pareto front is obtained, which contains the optimal solutions. The TOPSIS method is then employed to determine the most suitable solution among them. Return to Step 1 and update the trust network using the new preference matrix ${\boldsymbol{P}}_{e}^{{\left( {r+1} \right)}}={\left( {\tilde {p}_{{ij}}^{{e,\left( {r+1} \right)}}} \right)_{m \times n}}$. This results in a new HTN matrix ${\boldsymbol{H}}{\boldsymbol{T}}{{\boldsymbol{M}}^{\left( {r+1} \right)}}={\left( {HTD_{{eh}}^{{\left( {r+1} \right)}}} \right)_{k \times k}}$. After that, perform clustering again using Algorithm 1 to obtain the updated clustering result ${{\boldsymbol{G}}^{\left( {r+1} \right)}}=\left( {{G_1},{G_2}, \ldots ,{G_l}} \right)$.

*Step 6* Information aggregation. After the clustering is completed, update the weights of each DM according to Eq. (16), and aggregate the preference matrices of all DMs using Eq. (21). The group evaluation matrix generated is indicated as $${\boldsymbol{P}}_{G}^{{\left( {r+1} \right)}}=$$$${\left( {\tilde {p}_{{ij}}^{{G,\left( {r+1} \right)}}} \right)_{m \times n}}$$.

*Step 7* Alternative selection. At this stage, calculate evaluation values of each alternative based on the final group evaluation matrix together and attribute weights. Equation (2) provides the alternatives’ final scores, and the ranking results are determined accordingly, so as to identify the optimal solution.

## Case study

### Case description

With the intensification of global climate change and the accelerated transformation of the energy structure, renewable energy generation is gradually becoming a key initiative to promote green and low-carbon development. The scientific layout of wind and PV power plant stations not only affects energy utilization efficiency but also directly relates to regional economic development and ecological environment protection. The rational selection of locations for renewable energy power stations is of significant importance to achieve the strategic goals of carbon peak and carbon neutrality.

Suppose a local government needs to determine the best construction site of PV power plant among four alternatives ${\boldsymbol{X}}=\left\{ {{x_1},{x_2},{x_3},{x_4}} \right\}$, and has invited 20 experts ${\boldsymbol{V}}=\left\{ {{v_1},{v_2}, \ldots ,{v_{20}}} \right\}$ in closely related fields to participate in the DMP. These 20 experts evaluate the alternatives based on four attributes ${\boldsymbol{Y}}=\left\{ {{y_1},{y_2},{y_3},{y_4}} \right\}$, namely resource availability (${y_1}$, including solar resource abundance, sunshine duration, and terrain suitability), construction cost (${y_2}$, covering land acquisition, equipment investment, grid connection, and subsequent operation and maintenance costs), environmental impact (${y_3}$, involving impacts on the local ecosystem, land use, soil and water conservation, and landscape / visual effects), and social acceptance (${y_4}$, reflecting the level of local community support, employment opportunities, economic benefits, and policy coordination). The alternatives exhibit certain differences in terms of resource endowment, cost conditions, environmental constraints, and social acceptance.

To better express the uncertainty and preferences in the experts’ judgments, uncertain linguistic variables were used. The final site selection will be made through group consensus. Uncertain linguistic variables are defined as ***S*** = {*s*_0_, *s*_1_, *s*_2_, *s*_3_, *s*_4_, *s*_5_, *s*_6_, *s*_7_} = {very poor, poor, slightly poor, marginally poor, marginally good, slightly good, good, very good}.

Assume the unit cost for all DMs is 1, with attribute weights given by ***ω*** = [0.25, 0.20, 0.30, 0.25]. The consensus threshold is set as *η* = 0.90, the weight parameter as *β* = 0.50, and the similarity threshold limits are ${\sigma _U}=0.90$ and ${\sigma _L}=0.70$.

### Case analysis

*Step 1* Obtain initial information. The 20 DMs evaluated the four alternatives based on the four attributes. Their initial preference information and initial trust relationships were obtained, as shown in Table S1 (in the supplementary materials).

Firstly, use Eq. (5) to calculate the directed social matrix ${\boldsymbol{T}}{{\boldsymbol{M}}^{\left( 0 \right)}}={\left( {T{S_{eh}}} \right)_{20 \times 20}}$, as shown in Table S2 (in the supplementary materials).

$${\boldsymbol{T}}{{\boldsymbol{M}}^{\left( 0 \right)}}={\left[ {\begin{array}{*{20}{c}} 1&{\begin{array}{*{20}{l}} {0.45} \end{array}}& \ldots &{\begin{array}{*{20}{l}} {0.4} \end{array}} \\ {\begin{array}{*{20}{l}} {\begin{array}{*{20}{l}} {0.7} \end{array}} \end{array}}&{\begin{array}{*{20}{l}} 1 \end{array}}& \ldots &{\begin{array}{*{20}{l}} {0.45} \end{array}} \\ \vdots & \vdots & \ddots & \vdots \\ {\begin{array}{*{20}{l}} {0.6} \end{array}}&{\begin{array}{*{20}{l}} {0.45} \end{array}}& \ldots &{\begin{array}{*{20}{l}} 1 \end{array}} \end{array}} \right]_{20 \times 20}}$$

Then, by applying Eq. (10), preference similarity among DMs is derived, resulting in the similarity matrix ${\boldsymbol{S}}{{\boldsymbol{M}}^{\left( 0 \right)}}={\left( {SIM_{{eh}}^{{\left( 0 \right)}}} \right)_{20 \times 20}}$, which is presented in Table S3 (in the supplementary materials).

$${\boldsymbol{S}}{{\boldsymbol{M}}^{\left( 0 \right)}}={\left[ {\begin{array}{*{20}{c}} 1&{\begin{array}{*{20}{l}} {0.9286} \end{array}}& \ldots &{\begin{array}{*{20}{l}} {\begin{array}{*{20}{l}} {0.9107} \end{array}} \end{array}} \\ {\begin{array}{*{20}{l}} {\begin{array}{*{20}{l}} {0.9286} \end{array}} \end{array}}&{\begin{array}{*{20}{l}} 1 \end{array}}& \ldots &{\begin{array}{*{20}{l}} {\begin{array}{*{20}{l}} {0.8750} \end{array}} \end{array}} \\ \vdots & \vdots & \ddots & \vdots \\ {\begin{array}{*{20}{l}} {\begin{array}{*{20}{l}} {0.9107} \end{array}} \end{array}}&{\begin{array}{*{20}{l}} {\begin{array}{*{20}{l}} {0.875} \end{array}0} \end{array}}& \ldots &{\begin{array}{*{20}{l}} 1 \end{array}} \end{array}} \right]_{20 \times 20}}$$

Finally, use Eq. (12) to obtain the adjusted undirected social trust matrix ${\boldsymbol{A}}{\boldsymbol{T}}{{\boldsymbol{M}}^{\left( 0 \right)}}={\left( {ATS_{{eh}}^{{\left( 0 \right)}}} \right)_{20 \times 20}}$, as shown in Table S4 (in the supplementary materials).

$${\boldsymbol{A}}{\boldsymbol{T}}{{\boldsymbol{M}}^{\left( 0 \right)}}={\left[ {\begin{array}{*{20}{c}} 1&{\begin{array}{*{20}{l}} {0.45} \end{array}}& \ldots &{\begin{array}{*{20}{l}} {0.4} \end{array}} \\ {\begin{array}{*{20}{l}} {0.45} \end{array}}&{\begin{array}{*{20}{l}} 1 \end{array}}& \ldots &{\begin{array}{*{20}{l}} {0.45} \end{array}} \\ \vdots & \vdots & \ddots & \vdots \\ {\begin{array}{*{20}{l}} {0.4} \end{array}}&{\begin{array}{*{20}{l}} {0.45} \end{array}}& \ldots &{\begin{array}{*{20}{l}} 1 \end{array}} \end{array}} \right]_{20 \times 20}}$$

*Step 2* HTN construction. The matrix ${\boldsymbol{H}}{\boldsymbol{T}}{{\boldsymbol{M}}^{\left( 0 \right)}}={\left( {HTD_{{eh}}^{{\left( 0 \right)}}} \right)_{20 \times 20}}$ is obtained through Eq. (13) and reported in Table S5 (in the supplementary materials).

$${\boldsymbol{H}}{\boldsymbol{T}}{{\boldsymbol{M}}^{\left( 0 \right)}}={\left[ {\begin{array}{*{20}{c}} 1&{\begin{array}{*{20}{l}} {\begin{array}{*{20}{l}} {0.6893} \end{array}} \end{array}}& \ldots &{\begin{array}{*{20}{l}} {\begin{array}{*{20}{l}} {\begin{array}{*{20}{l}} {0.6554} \end{array}} \end{array}} \end{array}} \\ {\begin{array}{*{20}{l}} {\begin{array}{*{20}{l}} {\begin{array}{*{20}{l}} {0.6893} \end{array}} \end{array}} \end{array}}&{\begin{array}{*{20}{l}} 1 \end{array}}& \ldots &{\begin{array}{*{20}{l}} {\begin{array}{*{20}{l}} {0.6625} \end{array}} \end{array}} \\ \vdots & \vdots & \ddots & \vdots \\ {\begin{array}{*{20}{l}} {\begin{array}{*{20}{l}} {\begin{array}{*{20}{l}} {0.6554} \end{array}} \end{array}} \end{array}}&{\begin{array}{*{20}{l}} {0.6625} \end{array}}& \ldots &{\begin{array}{*{20}{l}} 1 \end{array}} \end{array}} \right]_{20 \times 20}}$$

*Step 3* Clustering process. 20 DMs are divided into subgroups by Algorithm 1 based on the HTN matrix. The clustering results are presented in Table 5.

**Table 5:** Initial clustering results.

Subgroup	Number of DMs	DMs
*G*_*1*_	3	*v*_11_, *v*_19_, *v*_2_
*G*_*2*_	5	*v*_1_, *v*_12_, *v*_20_, *v*_6_, *v*_9_
*G*_*3*_	5	*v*_10_, *v*_17_, *v*_3_, *v*_5_, *v*_7_
*G*_*4*_	7	*v*_13_, *v*_14_, *v*_15_, *v*_16_, *v*_18_, *v*_4_, *v*_8_

*Step 4* Consensus measurement. Weights are assigned to individual DMs, subgroups, and members within subgroups according to Eqs. (16)–(18). Then, consensus levels for DMs, subgroups, and the group as a whole are calculated by Eqs. (19), (22), and (23). The outcomes are presented in Table 6.

**Table 6:** Initial subgroup clustering weight and consensus level results.

Subgroup	DM	ICD	Intra-cluster weight	Cluster weight	CL
*G*_*1*_	*v*_*11*_	0.8951	0.3475	0.1496	0.8725
	*v*_*19*_	0.8556	0.3263		
	*v*_*2*_	0.8651	0.3262		
*G*_*2*_	*v*_*1*_	0.8501	0.1999	0.2488	0.8741
	*v*_*12*_	0.9065	0.1955		
	*v*_*20*_	0.8790	0.2025		
	*v*_*6*_	0.8655	0.1988		
	*v*_*9*_	0.8701	0.2033		
*G*_*3*_	*v*_*10*_	0.8687	0.1933	0.2480	0.8709
	*v*_*17*_	0.8912	0.2021		
	*v*_*3*_	0.9085	0.2044		
	*v*_*5*_	0.8674	0.1956		
	*v*_*7*_	0.8783	0.2046		
*G*_*4*_	*v*_*13*_	0.9023	0.1505	0.3536	0.8748
	*v*_*14*_	0.8762	0.1461		
	*v*_*15*_	0.8450	0.1370		
	*v*_*16*_	0.8614	0.1406		
	*v*_*18*_	0.8877	0.1459		
	*v*_*4*_	0.8618	0.1395		
	*v*_*8*_	0.8855	0.1405		

At this point, $GC{L^{\left( 0 \right)}}=0.8733<\eta$ and it is lower than the preset threshold *η*. Therefore, the process moves to the feedback mechanism stage of the CRP.

**Fig. 2:**
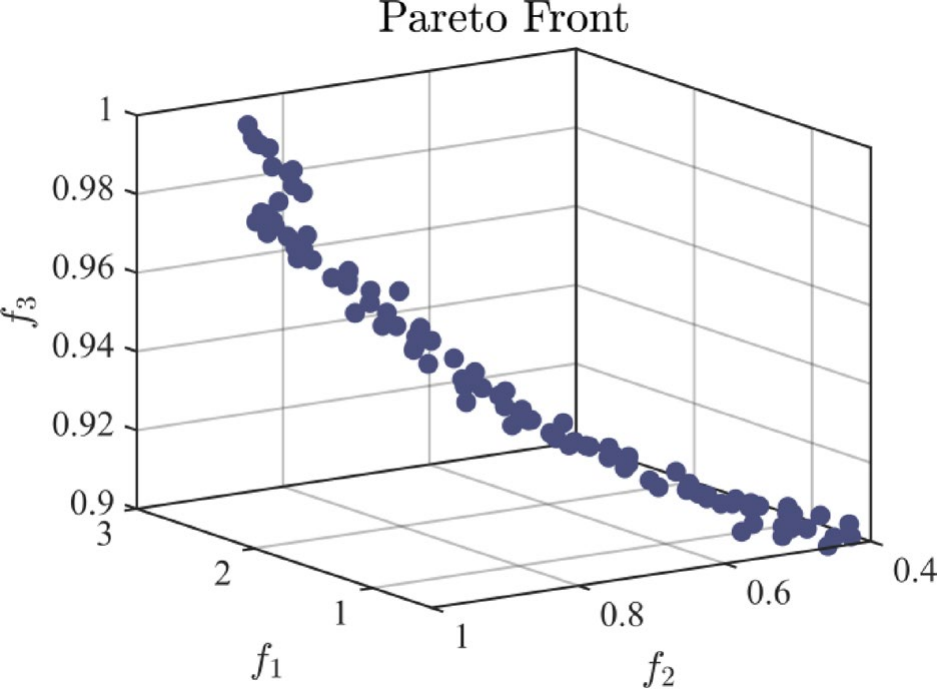
Pareto optimal solutions.

*Step 5* Feedback mechanism. By solving Model M3, Pareto optimal solutions are produced, which helps achieve group consensus. The outcomes are illustrated in Table 7, and Fig. 2 reports several Pareto optimal results.

**Table 7:** Representative Pareto optimal solutions.

Pareto	Cost	Fairness	GCL
*f*(*x*_1_)	1.4309	0.7909	0.9446
*f*(*x*_2_)	1.7422	0.9043	0.9597
*f*(*x*_3_)	1.5060	0.8075	0.9478
*f*(*x*_4_)	1.9774	0.9252	0.9715
*f*(*x*_5_)	1.5537	0.8547	0.9508
*f*(*x*_6_)	1.4894	0.8452	0.9477
*f*(*x*_7_)	0.8969	0.6384	0.9182
*f*(*x*_8_)	1.7258	0.8636	0.9588
*f*(*x*_9_)	0.9580	0.6625	0.9214
*f*(*x*_10_)	1.6071	0.8425	0.9533

The TOPSIS is applied to sort Pareto-optimal solutions. As a result, *f*(*x*_2_) is identified as the optimal solution, with its adjustment parameters being ${\bar {{\boldsymbol{o}}}_e}$ = [0.6891, 0.5025, 0.6311, 0.6795, 0.5515, 0.7746, 0.6501, 0.7133, 0.7618, 0.6291, 0.6811, 0.9729, 0.6706, 0.5412, 0.6196, 0.8256, 0.6633, 0.6870, 0.6619, 0.9315]. Then, return to Step 1 and adjust the trust network in combination with the updated preference matrix to obtain the new HTN matrix, as displayed in Table S6 (in the supplementary materials).

$${\boldsymbol{H}}{\boldsymbol{T}}{{\boldsymbol{M}}^{\left( 1 \right)}}={\left[ {\begin{array}{*{20}{c}} 1&{\begin{array}{*{20}{l}} {\begin{array}{*{20}{l}} {0.6441} \end{array}} \end{array}}& \ldots &{\begin{array}{*{20}{l}} {\begin{array}{*{20}{l}} {\begin{array}{*{20}{l}} {0.6469} \end{array}} \end{array}} \end{array}} \\ {\begin{array}{*{20}{l}} {\begin{array}{*{20}{l}} {\begin{array}{*{20}{l}} {0.6441} \end{array}} \end{array}} \end{array}}&{\begin{array}{*{20}{l}} 1 \end{array}}& \ldots &{\begin{array}{*{20}{l}} {\begin{array}{*{20}{l}} {0.6699} \end{array}} \end{array}} \\ \vdots & \vdots & \ddots & \vdots \\ {\begin{array}{*{20}{l}} {\begin{array}{*{20}{l}} {\begin{array}{*{20}{l}} {0.6469} \end{array}} \end{array}} \end{array}}&{\begin{array}{*{20}{l}} {0.6699} \end{array}}& \ldots &{\begin{array}{*{20}{l}} 1 \end{array}} \end{array}} \right]_{20 \times 20}}$$

Clustering is performed again using Algorithm 1. The new clustering results are $$G_{1}^{{\left( 1 \right)}}=\left\{ {{v_1},{v_{11}},{v_{19}},{v_2}} \right\}$$, $$G_{2}^{{\left( 1 \right)}}=\left\{ {{v_{10}},{v_{13}},{v_{14}},{v_{15}},{v_{16}},{v_{17}},{v_{18}}{v_3},{v_4},{v_5},{v_7},{v_8}} \right\}$$, and $$G_{3}^{{\left( 1 \right)}}=\left\{ {{v_{12}},} \right.$$$$\left. {{v_{20}},{v_6},{v_9}} \right\}$$.

*Step 6* Information aggregation. Once clustering is completed, updated DM weights can be obtained according to Eq. (16). Subsequently, the preference matrices of all DMs are aggregated by Eq. (21) to generate the final group evaluation matrix as follows:

$${\boldsymbol{P}}_{G}^{{\left( 1 \right)}}={\left( {\begin{array}{*{20}{c}} {\left( {{s_{3.6410}},{s_{5.0079}}} \right)}&{\left( {{s_{2.1024}},{s_{3.4206}}} \right)}&{\left( {{s_{1.9217}},{s_{3.2582}}} \right)}&{\left( {{s_{3.0565}},s{}_{{4.2501}}} \right)} \\ {\left( {{s_{3.4628}},{s_{4.9462}}} \right)}&{\left( {{s_{1.9415}},{s_{3.7999}}} \right)}&{\left( {{s_{3.2918}},{s_{5.2499}}} \right)}&{\left( {{s_{3.7731}},{s_{5.6883}}} \right)} \\ {\left( {{s_{4.0879}},{s_{5.5004}}} \right)}&{\left( {{s_{3.1247}},{s_{4.1720}}} \right)}&{\left( {{s_{2.9454}},{s_{4.1750}}} \right)}&{\left( {{s_{3.1169}},{s_{4.4613}}} \right)} \\ {\left( {{s_{3.0427}},{s_{4.5007}}} \right)}&{\left( {{s_{2.0860}},{s_{3.3851}}} \right)}&{\left( {{s_{1.7211}},{s_{3.0694}}} \right)}&{\left( {{s_{2.6989}},{s_{4.3518}}} \right)} \end{array}} \right)_{4 \times 4}}$$

*Step 7* Alternative selection. Finally, the evaluation intervals of the four alternatives are reckoned according to the group evaluation matrix and attribute weights, that is$${\tilde {p}_1}=\left[ {2.6713,3.9761} \right]$$, $${\tilde {p}_2}=\left[ {3.1848,4.9936} \right]$$, $${\tilde {p}_3}=\left[ {3.3098,4.5773} \right]$$, $${\tilde {p}_4}=\left[ {2.3689,3.8110} \right]$$. With Eq. (2), final scores of the four alternatives are $$DI\left( {{{\tilde {p}}_1}} \right)={\mathrm{0}}{\mathrm{.4757}}$$, $$DI\left( {{{\tilde {p}}_2}} \right)={\mathrm{0}}{\mathrm{.5788}}$$, $$DI\left( {{{\tilde {p}}_3}} \right)={\mathrm{0}}{\mathrm{.5613}}$$, $$DI\left( {{{\tilde {p}}_1}} \right)=$$$${\mathrm{0}}{\mathrm{.4438}}$$,and then the ranking is established as $${x_2}>{x_3}>{x_1}>{x_4}$$, showing that $${x_2}$$ is the best option.

## Sensitivity and comparison analysis

### Sensitivity analysis

#### Sensitivity analysis of the consensus threshold

**Fig. 3:**
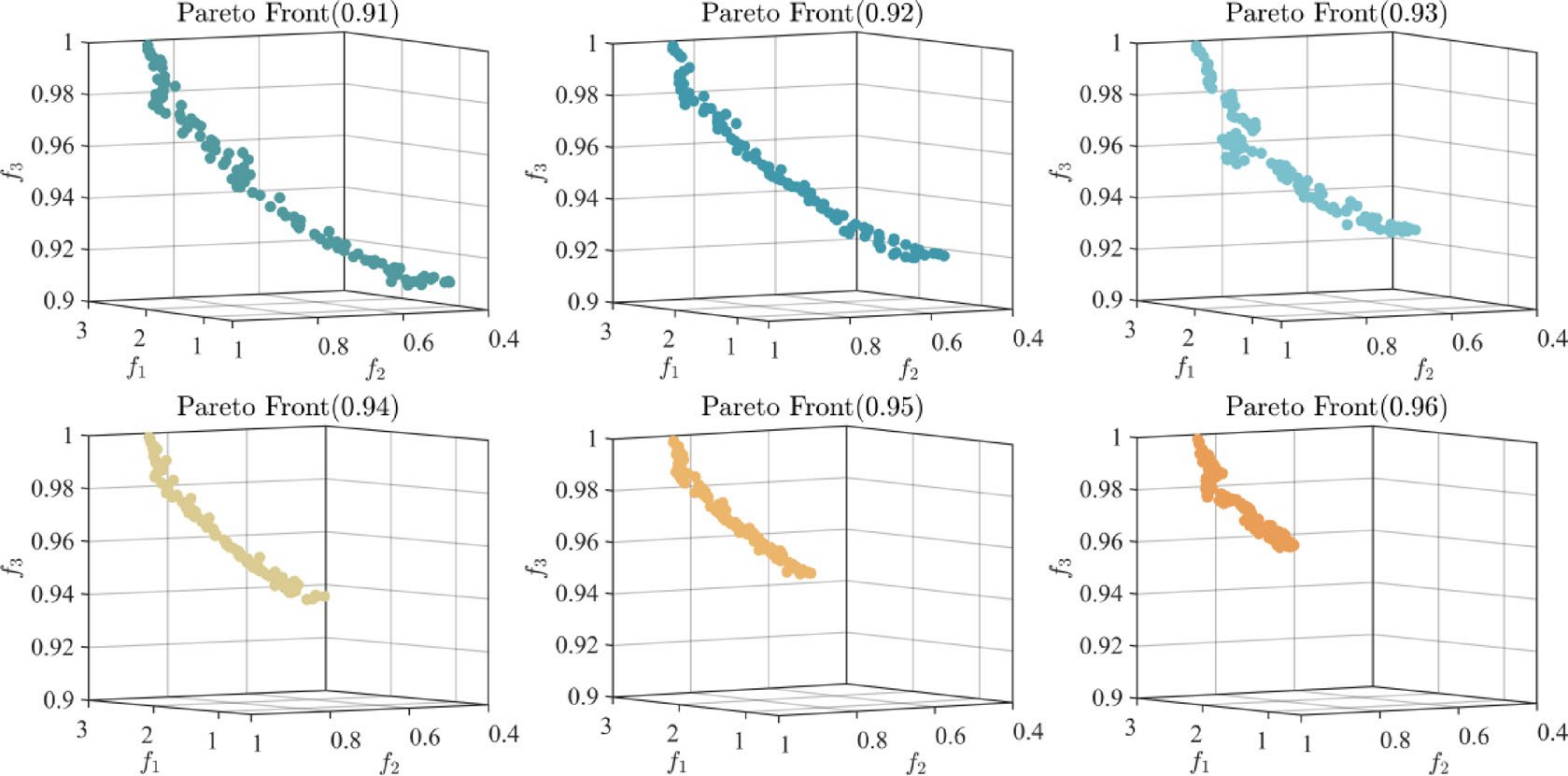
Pareto optimal solutions obtained under different thresholds.

To further examine how varying consensus thresholds influence cost, fairness, and GCL, *η* was gradually tuned from 0.91 to 0.96. Figure 3 displays the Pareto optimal solutions obtained under these different thresholds.

The findings show that consensus thresholds have a direct influence on cost, fairness, and GCL in GDM. A lower consensus threshold helps reduce decision costs but may compromise fairness and consensus level. However, a higher consensus threshold improves fairness and consensus level at the expense of increased costs. Therefore, in practical DMP, a careful balance in selecting the consensus threshold is necessary to ensure a reasonable trade-off among cost, fairness, and consensus level. In situations with limited resources, DMs should determine a consensus threshold that fits the context, so as to reduce costs while maintaining fairness and consensus.

#### Sensitivity analysis of the weight parameter *β*

**Fig. 4:**
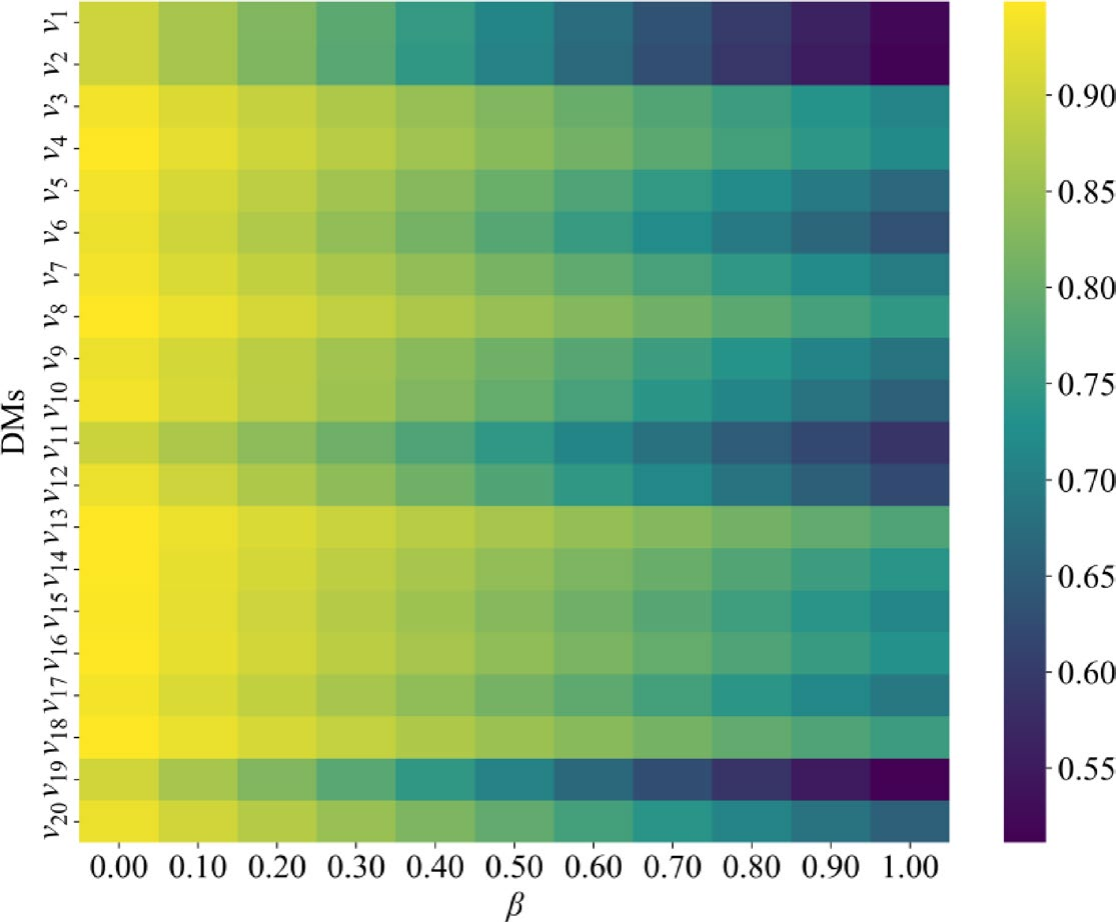
Heatmap of HTD values at different *β* values.

By adjusting *β*, the weight parameter balances trust degrees and preference similarity among DMs in the HTN, which determines the HTD value. A heatmap is then applied to evaluate HTD values for DMs under different *β* conditions, as presented in Fig. 4.

The results show that when *β* is close to 0, the results are mainly influenced by preference similarity. When *β* is close to 1, the results are primarily determined by trust relationships. Additionally, the sensitivity of DMs to changes in *β* varies. For example, the HTD values of DMs *v*_19_, *v*_1_, and *v*_2_ fluctuate significantly under different *β* values, indicating higher sensitivity and a greater impact on how the HTN is built. In contrast, the HTD values of DMs *v*_13_, *v*_18_, and *v*_8_ remain relatively stable with minimal changes, demonstrating more consistent performance in the HTN. Therefore, the selection of *β* should be based on actual requirements to optimize the weight distribution.

#### Sensitivity analysis of the attribute weights

**Fig. 5:**
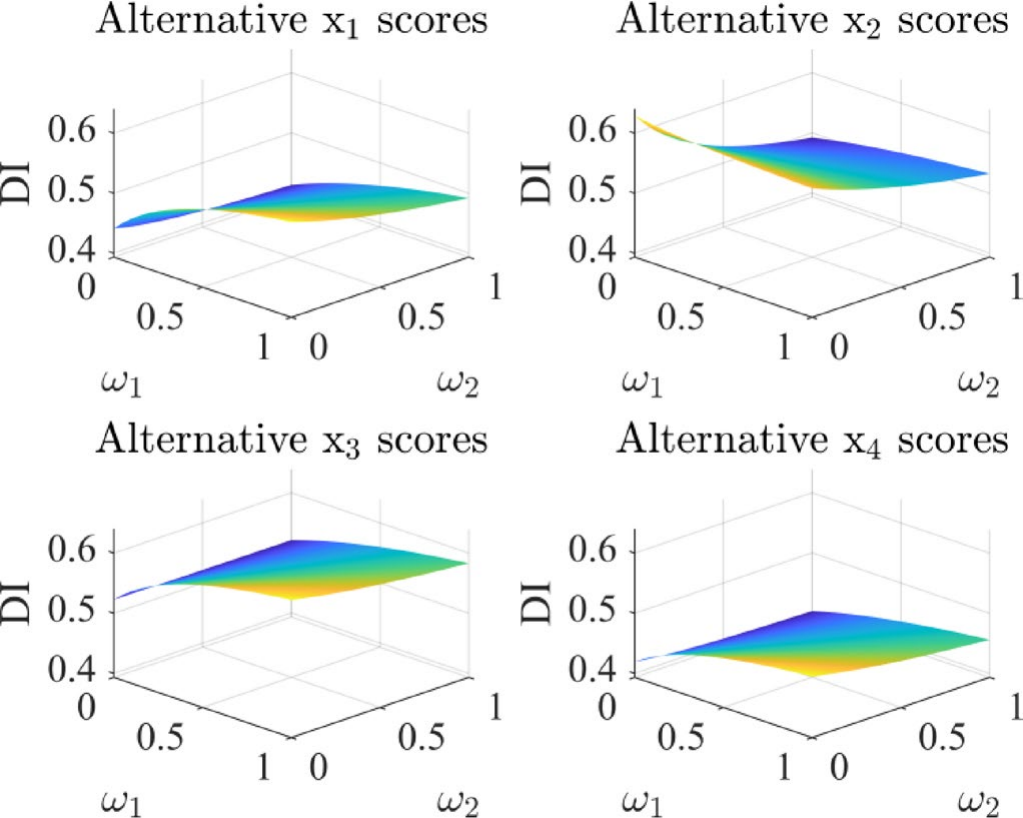
The scores of alternatives with different *ω*_1_, *ω*_2_ values.

**Fig. 6:**
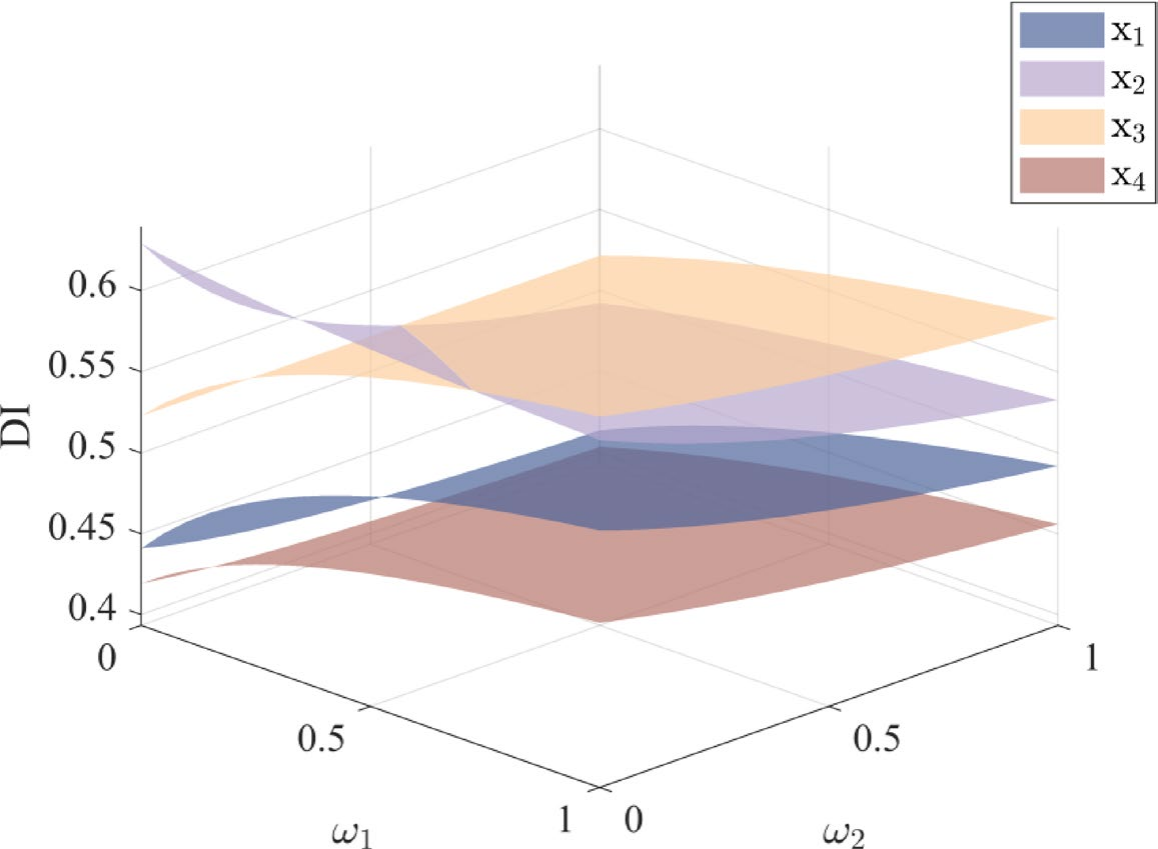
Comparison of the scores of each alternative under different values of *ω*_1_ and *ω*_2_.

**Fig. 7:**
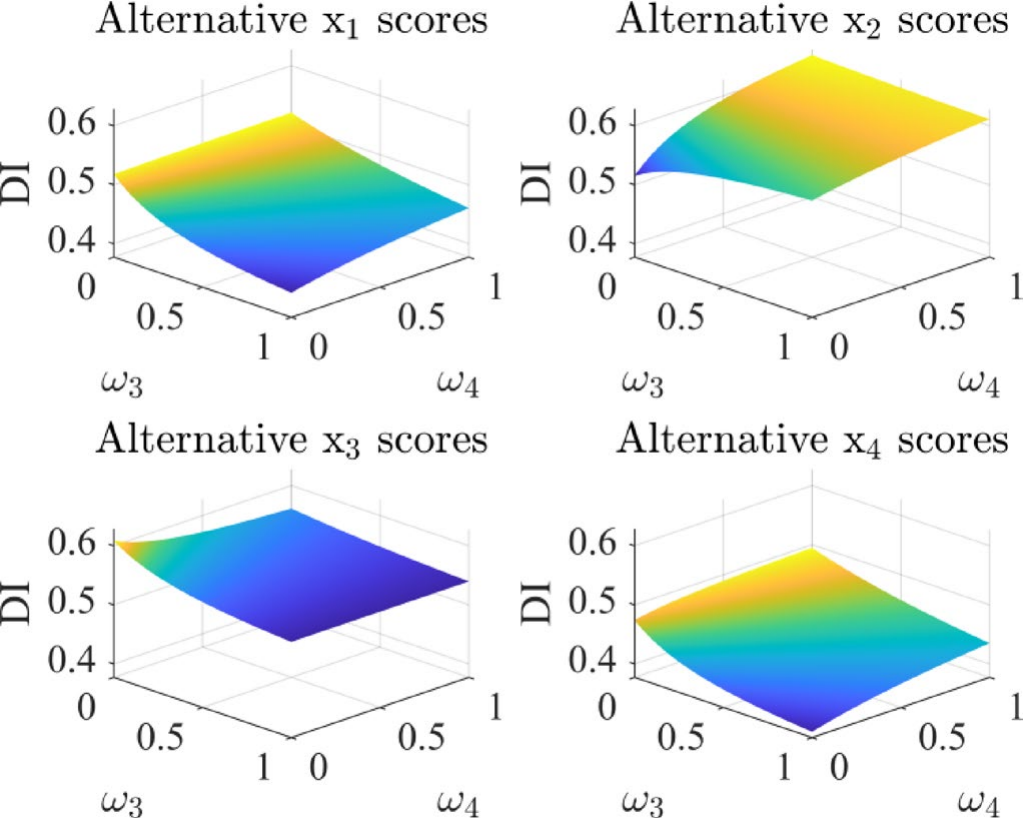
The scores of alternatives with different *ω*_3_, *ω*_4_ values.

**Fig. 8:**
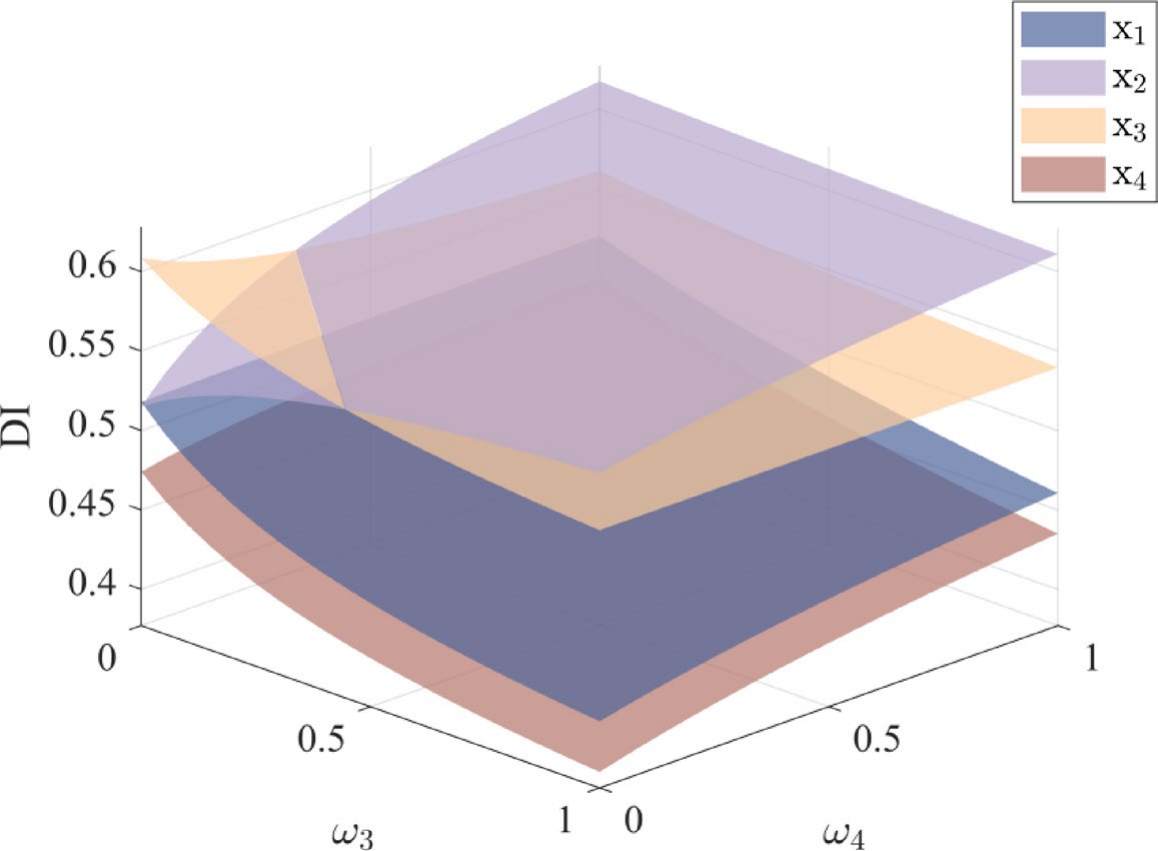
Comparison of the scores of each alternative under different values of *ω*_3_ and *ω*_4_.

By varying attribute weights, this section evaluates their influence on alternative scores and rankings. The results are presented in Figs. 5, 6, 7 and 8.

The results show that different combinations of attribute weights pose some impact on the alternative scores. When the weights of attributes *ω*_1_ and *ω*_2_ change, the rankings of alternatives ${x_2}$ and ${x_3}$ show some variation. In most cases, alternative ${x_3}$ ranks higher than ${x_2}$, while the rankings of alternatives ${x_1}$ and ${x_4}$ remain stable. Similarly, when the weights of attributes *ω*_3_ and *ω*_4_ change, alternative ${x_2}$ is superior to ${x_3}$ in most situations. Therefore, when setting attribute weights, DMs should give priority to factors that strongly influence the results, thereby enhancing the selection process.

### Comparison analysis

To demonstrate the method’s validity and robustness, a qualitative comparison is carried out focusing on clustering approaches and optimization models.

#### Comparison of different clustering methods

A comparison among various clustering methods is summarized in Table 8. In LGDM, whether clustering is applied affects the improvement of the GCL. However, the majority of existing work take a single perspective of similarity preference or trust relationships as the basis of clustering. Zheng et al.^18^ introduced a clustering method that integrates hesitation and fuzziness, relying on preference similarity and compatibility to enhance subgroup cohesion. Meanwhile, Meng et al.^17^ designated the expert with the highest trust as the subgroup center and developed a clustering strategy that incorporates trust density and opinion similarity to divide the group into subgroups. As shown in Table 9, the clustering results based only on preference similarity generally show unbalanced subgroup sizes. In contrast, after introducing both trust relationships and preference similarity in the clustering process, the subgroup division obtained in this paper is more balanced in size. This provides a more reasonable subgroup structure as a basis for the subsequent CRP. Thus, incorporating both preference similarity and trust relationships into clustering enables a more thorough representation of the interconnections among DMs.

**Table 8:** Comparison with different clustering methods.

Method	Decision problem	Clustering method	Clustering basis	Trust relationship
Lu et al.^62^	LGDM	K-means	Trust relationship	Static trust
Zheng et al.^18^^18^	LGDM	Preference similarity and compatibility-based clustering	Preference similarity and compatibility	×
Wu et al.^19^	LGDM	Random grouping	Preference similarity	×
Xing et al.^25^	SNGDM	×	×	Dynamic trust
Shen et al.^59^	LGDM	Consensus level maximization-based clustering	Consensus level	Static trust
Meng et al.^17^	LGDM	Trust density and opinion similarity-based clustering	Trust density, preference similarity	Dynamic trust
Proposed method	LGDM	Louvain algorithm	Trust density, preference similarity	Dynamic trust

**Table 9:** Comparative analysis of clustering results.

	Considering both trust relationships and preference similarity	Considering only preference similarity
G_1_	v_11_, v_19_, v_2_	v_1_, v_12_, v_18_, v_4_, v_5_, v_6_, v_8_
*G*_*2*_	*v*_1_, *v*_12_, *v*_20_, *v*_6_, *v*_9_	*v*_10_, *v*_11_, *v*_13_, *v*_14_, *v*_15_, *v*_16_, *v*_17_, *v*_19_, *v*_2_, *v*_20_, *v*_3_, *v*_7_, *v*_9_
*G*_*3*_	*v*_10_, *v*_17_, *v*_3_, *v*_5_, *v*_7_	—
*G*_*4*_	*v*_13_, *v*_14_, *v*_15_, *v*_16_, *v*_18_, *v*_4_, *v*_8_	—

Many scholars use static trust relationships when building social networks, without considering their dynamic evolution. This limits the analysis to a static trust network environment. However, trust relationships serve as the foundation for evaluating a DM’s influence. A dynamic trust network provides a more precise depiction of DMs’ dynamic weights, thereby influencing the subgroup weights. Some studies have explored this issue. Wu et al.^19^ dynamically adjusted subgroup weights based on subgroup consensus levels, while Xing et al.^25^ introduced dynamic trust into LGDM and determined experts’ weights by a maximum entropy model which incorporates individual interaction relationships into fairness of dynamic trust relationships.

Therefore, grounded in the prior qualitative comparison, the proposed framework establishes an HTN by combining preference similarity with trust relations, which then functions as the clustering basis. Unlike methods that only examine static trust relationships, this study adjusts the evaluation matrices through a feedback mechanism and uses the updated preference similarity to modify the trust relationships. This enables re-clustering and the dynamic determination of DMs’ weights, thus better aligning with real-world DMP.

#### Comparison of different optimization models

An analysis contrasting multiple optimization models is displayed in Table 10. Zhang et al.^46^ proposed a minimum cost consensus model that seeks a balance between cost and the consensus level. Cheng et al.^63^ developed a model which incorporates individual tolerance and limited compromise behavior to maximize satisfaction under a constrained budget. Also considering limited compromise behavior, Xu and Xu^13^ determined a budget limit by a minimum cost consensus model and then integrated it into a maximum satisfaction consensus model (MSCM). However, the above studies did not address the issue of fairness in the DMP. Therefore, Shen et al.^59^ constructed a MOOCM targeting minimum cost and maximum fairness, thereby achieving a better trade-off between these two objectives during the LGDM process (MCMF-MO-LGDM).

**Table 10:** Comparison with different clustering methods.

Reference	Cost	Fairness	Consensus level	Optimization model
Zhang et al.^46^	√	×	√	MCCM
Cheng et al.^63^	√	×	√	MSCM
Xu and Xu ^13^	√	×	√	MSCM
Shen et al.^59^	√	√	√	MCMF-MO-LGDM
Proposed method	√	√	√	MCMFMCL-MOOCM

Building on their work, our study introduces the maximization of consensus level as a third objective, extending the model from a two-dimensional to a three-dimensional optimization framework. This extension enhances the model’s adaptability and practical utility in real-world DMP.

## Conclusion

A MOOCM based on dynamic trust networks is proposed for LGDM problems. Firstly, an HTN is established by combining preference similarity with trust relations, and DMs are grouped using the Louvain algorithm to lower decision complexity. Secondly, a MOOCM is designed, aiming at minimum cost, maximum fairness and maximum consensus level. By generating Pareto optimal solutions, the model provides DMs with a broader decision space. Then, through dynamic trust network updates and secondary clustering after the CRP, the dynamic weights of DMs are further determined, ensuring more reasonable decision outcomes. Finally, the approach is finally examined in a PV power plant site selection problem, where sensitivity and comparative analyses confirm its effectiveness and feasibility. The main conclusions can be summarized as follows:

1. A Louvain algorithm utilizing an HTN is proposed. By combining preference similarity with trust relationships to construct the HTN for clustering, the method effectively reduces the dimensionality of LGDM, thus enhancing both the efficiency and rationality of the process. In the case study, 20 DMs are first clustered into four subgroups, which provides the basis for the subsequent CRP.
2. A MOOCM is proposed, driven by the goals of minimizing cost, maximizing fairness, and maximizing the GCL. The model produces Pareto optimal solutions, providing DMs with diverse options and enabling them to achieve a balance among multiple objectives. Consequently, it supports more scientific and impartial decisions. In the case study, a representative Pareto solution achieves GCL = 0.9597 with Cost = 1.7422 and Fairness = 0.9043, demonstrating the effectiveness of the proposed model.
3. A dynamic trust network updating mechanism is introduced. During the consensus feedback process, preference similarity is recalculated based on adjusted preferences to update the HTN, and a second clustering is performed to dynamically adjust DMs’ weights. In the case study, the number of subgroups is adjusted from four to three after updating the HTN, indicating that the proposed mechanism can better capture the evolution of trust relationships and improve the rationality of weight allocation.

However, this paper is not without limitations. For instance, it presumes complete trust relationships among DMs, whereas in practice, these relationships are often incomplete or uncertain. Future research can further explore methods to complete incomplete trust networks, so as to improve the applicability and practical value of the proposed method.

## Supplementary Information

Below is the link to the electronic supplementary material.

Supplementary Material 1

## Data Availability

All data supporting the findings of this study are available within the article and its Supplementary Information.
