# Supplementary material for: A multi-objective optimization consensus model for large-scale group decision-making considering dynamic social networks
Source: Sci Rep. 2026 Apr 1;16:15314. doi: 10.1038/s41598-026-45239-0 (PMC13180972; doi:10.1038/s41598-026-45239-0)
Supplement: Supplementary file 1 — Supplementary Material 1 [file 41598_2026_45239_MOESM1_ESM.docx]

The initial decision-making information:

$P_{1}^{\left(0\right)}=\left(\begin{matrix} \left(4,7\right) & \left(2,2\right) & \left(2,3\right) & \left(2,3\right) \\ \left(4,6\right) & \left(3,5\right) & \left(3,5\right) & \left(0,2\right) \\ \left(4,4\right) & \left(2,3\right) & \left(3,3\right) & \left(1,2\right) \\ \left(2,3\right) & \left(2,2\right) & \left(1,2\right) & \left(4,4\right) \end{matrix}\right)$, $P_{2}^{\left(0\right)}=\left(\begin{matrix} \left(4,5\right) & \left(1,2\right) & \left(0,3\right) & \left(4,6\right) \\ \left(0,3\right) & \left(4,7\right) & \left(4,7\right) & \left(5,7\right) \\ \left(5,6\right) & \left(4,4\right) & \left(3,5\right) & \left(4,6\right) \\ \left(1,3\right) & \left(4,4\right) & \left(2,2\right) & \left(2,2\right) \end{matrix}\right)$,

$P_{3}^{\left(0\right)}=\left(\begin{matrix} \left(3,5\right) & \left(3,6\right) & \left(3,4\right) & \left(1,2\right) \\ \left(3,4\right) & \left(1,2\right) & \left(3,6\right) & \left(3,6\right) \\ \left(4,7\right) & \left(5,6\right) & \left(3,6\right) & \left(2,4\right) \\ \left(4,4\right) & \left(2,6\right) & \left(2,4\right) & \left(1,3\right) \end{matrix}\right)$, $P_{4}^{\left(0\right)}=\left(\begin{matrix} \left(4,6\right) & \left(2,4\right) & \left(0,2\right) & \left(4,5\right) \\ \left(5,7\right) & \left(2,2\right) & \left(5,7\right) & \left(4,7\right) \\ \left(3,6\right) & \left(3,5\right) & \left(2,2\right) & \left(3,4\right) \\ \left(1,2\right) & \left(3,3\right) & \left(2,5\right) & \left(3,5\right) \end{matrix}\right)$,

$P_{5}^{\left(0\right)}=\left(\begin{matrix} \left(2,4\right) & \left(2,3\right) & \left(2,2\right) & \left(3,4\right) \\ \left(4,6\right) & \left(0,2\right) & \left(3,5\right) & \left(5,7\right) \\ \left(4,5\right) & \left(2,3\right) & \left(0,2\right) & \left(1,1\right) \\ \left(3,3\right) & \left(2,2\right) & \left(2,3\right) & \left(2,6\right) \end{matrix}\right)$, $P_{6}^{\left(0\right)}=\left(\begin{matrix} \left(3,5\right) & \left(4,5\right) & \left(1,1\right) & \left(3,4\right) \\ \left(4,5\right) & \left(2,4\right) & \left(6,7\right) & \left(7,7\right) \\ \left(5,7\right) & \left(2,3\right) & \left(3,3\right) & \left(5,6\right) \\ \left(2,3\right) & \left(1,3\right) & \left(1,2\right) & \left(2,5\right) \end{matrix}\right)$,

$P_{7}^{\left(0\right)}=\left(\begin{matrix} \left(4,4\right) & \left(2,2\right) & \left(0,4\right) & \left(5,7\right) \\ \left(3,4\right) & \left(1,3\right) & \left(2,5\right) & \left(4,4\right) \\ \left(4,6\right) & \left(3,6\right) & \left(3,5\right) & \left(3,3\right) \\ \left(4,5\right) & \left(2,2\right) & \left(3,5\right) & \left(4,6\right) \end{matrix}\right)$, $P_{8}^{\left(0\right)}=\left(\begin{matrix} \left(4,5\right) & \left(2,6\right) & \left(1,2\right) & \left(2,5\right) \\ \left(4,5\right) & \left(0,3\right) & \left(4,5\right) & \left(4,6\right) \\ \left(3,3\right) & \left(3,3\right) & \left(3,3\right) & \left(0,2\right) \\ \left(2,3\right) & \left(2,5\right) & \left(3,3\right) & \left(3,5\right) \end{matrix}\right)$,

$P_{9}^{\left(0\right)}=\left(\begin{matrix} \left(5,5\right) & \left(2,2\right) & \left(0,4\right) & \left(3,5\right) \\ \left(3,7\right) & \left(3,4\right) & \left(2,5\right) & \left(4,7\right) \\ \left(6,6\right) & \left(3,5\right) & \left(3,7\right) & \left(4,4\right) \\ \left(4,7\right) & \left(4,6\right) & \left(0,2\right) & \left(3,4\right) \end{matrix}\right)$, $P_{10}^{\left(0\right)}=\left(\begin{matrix} \left(2,4\right) & \left(4,6\right) & \left(3,4\right) & \left(3,4\right) \\ \left(4,5\right) & \left(5,7\right) & \left(3,5\right) & \left(3,7\right) \\ \left(4,6\right) & \left(4,5\right) & \left(4,4\right) & \left(4,6\right) \\ \left(4,5\right) & \left(2,2\right) & \left(4,4\right) & \left(5,5\right) \end{matrix}\right)$

$P_{11}^{\left(0\right)}=\left(\begin{matrix} \left(4,5\right) & \left(1,2\right) & \left(5,6\right) & \left(4,6\right) \\ \left(2,5\right) & \left(3,5\right) & \left(3,4\right) & \left(4,5\right) \\ \left(3,4\right) & \left(3,5\right) & \left(4,7\right) & \left(3,5\right) \\ \left(2,4\right) & \left(2,5\right) & \left(1,2\right) & \left(2,3\right) \end{matrix}\right)$, $P_{12}^{\left(0\right)}=\left(\begin{matrix} \left(4,7\right) & \left(2,4\right) & \left(1,1\right) & \left(4,4\right) \\ \left(3,6\right) & \left(0,2\right) & \left(3,6\right) & \left(5,6\right) \\ \left(5,5\right) & \left(3,4\right) & \left(3,4\right) & \left(5,5\right) \\ \left(4,6\right) & \left(1,2\right) & \left(0,2\right) & \left(2,4\right) \end{matrix}\right)$,

$P_{13}^{\left(0\right)}=\left(\begin{matrix} \left(3,4\right) & \left(1,2\right) & \left(4,4\right) & \left(4,4\right) \\ \left(4,5\right) & \left(1,5\right) & \left(4,4\right) & \left(5,7\right) \\ \left(4,6\right) & \left(3,4\right) & \left(3,3\right) & \left(4,4\right) \\ \left(3,5\right) & \left(1,2\right) & \left(2,3\right) & \left(3,4\right) \end{matrix}\right)$, $P_{14}^{\left(0\right)}=\left(\begin{matrix} \left(4,5\right) & \left(0,2\right) & \left(2,3\right) & \left(4,5\right) \\ \left(4,5\right) & \left(2,2\right) & \left(4,7\right) & \left(3,4\right) \\ \left(4,6\right) & \left(2,3\right) & \left(4,5\right) & \left(4,5\right) \\ \left(4,7\right) & \left(1,4\right) & \left(2,2\right) & \left(2,4\right) \end{matrix}\right)$

$P_{15}^{\left(0\right)}=\left(\begin{matrix} \left(4,4\right) & \left(3,5\right) & \left(2,2\right) & \left(1,3\right) \\ \left(3,4\right) & \left(0,1\right) & \left(1,5\right) & \left(3,5\right) \\ \left(3,4\right) & \left(4,4\right) & \left(4,4\right) & \left(4,4\right) \\ \left(5,6\right) & \left(5,5\right) & \left(2,5\right) & \left(3,5\right) \end{matrix}\right)$, $P_{16}^{\left(0\right)}=\left(\begin{matrix} \left(4,5\right) & \left(4,4\right) & \left(4,5\right) & \left(2,3\right) \\ \left(4,4\right) & \left(2,7\right) & \left(3,3\right) & \left(4,7\right) \\ \left(4,6\right) & \left(1,3\right) & \left(2,3\right) & \left(4,6\right) \\ \left(3,5\right) & \left(1,2\right) & \left(2,2\right) & \left(2,3\right) \end{matrix}\right)$,

$P_{17}^{\left(0\right)}=\left(\begin{matrix} \left(5,7\right) & \left(1,2\right) & \left(3,3\right) & \left(4,4\right) \\ \left(3,6\right) & \left(2,4\right) & \left(3,5\right) & \left(2,4\right) \\ \left(4,5\right) & \left(3,3\right) & \left(3,5\right) & \left(3,6\right) \\ \left(3,5\right) & \left(3,4\right) & \left(2,4\right) & \left(3,5\right) \end{matrix}\right)$, $P_{18}^{\left(0\right)}=\left(\begin{matrix} \left(2,5\right) & \left(4,5\right) & \left(1,3\right) & \left(4,5\right) \\ \left(4,4\right) & \left(3,4\right) & \left(4,4\right) & \left(4,4\right) \\ \left(5,6\right) & \left(3,3\right) & \left(3,4\right) & \left(2,6\right) \\ \left(3,4\right) & \left(1,2\right) & \left(1,3\right) & \left(4,5\right) \end{matrix}\right)$,

$P_{19}^{\left(0\right)}=\left(\begin{matrix} \left(4,6\right) & \left(2,2\right) & \left(3,4\right) & \left(0,1\right) \\ \left(4,5\right) & \left(1,1\right) & \left(4,7\right) & \left(3,6\right) \\ \left(5,6\right) & \left(4,6\right) & \left(3,6\right) & \left(5,6\right) \\ \left(4,5\right) & \left(2,3\right) & \left(1,3\right) & \left(4,7\right) \end{matrix}\right)$, $P_{20}^{\left(0\right)}=\left(\begin{matrix} \left(4,4\right) & \left(0,2\right) & \left(1,5\right) & \left(4,5\right) \\ \left(4,4\right) & \left(3,5\right) & \left(3,5\right) & \left(4,6\right) \\ \left(4,6\right) & \left(4,4\right) & \left(3,3\right) & \left(2,5\right) \\ \left(2,5\right) & \left(0,2\right) & \left(0,2\right) & \left(0,2\right) \end{matrix}\right)$.

**Table** **S1.** The initial trust network relations.

|  | *v*_1_ | | *v*_2_ | | *v*_3_ | | *v*_4_ | | *v_5_* | | *v*_6_ | | *v*_7_ | | *v*_8_ | | *v*_9_ | | *v*_10_ | | *v*_11_ | | *v*_12_ | | *v*_13_ | | *v*_14_ | | *v*_15_ | | *v*_16_ | | *v*_17_ | | *v*_18_ | | *v*_19_ | | *v*_20_ | |
| --- | --- | --- | --- | --- | --- | --- | --- | --- | --- | --- | --- | --- | --- | --- | --- | --- | --- | --- | --- | --- | --- | --- | --- | --- | --- | --- | --- | --- | --- | --- | --- | --- | --- | --- | --- | --- | --- | --- | --- | --- |
|  | t | d | t | d | t | d | t | d | t | d | t | d | t | d | t | d | t | d | t | d | t | d | t | d | t | d | t | d | t | d | t | d | t | d | t | d | t | d | t | d |
| *v*_1_ | 1 | 0 | 0.3 | 0.4 | 0.4 | 0.2 | 0.6 | 0.3 | 0.4 | 0.3 | 0.5 | 0.4 | 0.4 | 0.5 | 0.2 | 0.6 | 0.8 | 0.2 | 0.7 | 0.2 | 0.8 | 0.2 | 0.4 | 0.4 | 0.5 | 0.3 | 0.3 | 0.6 | 0.7 | 0.3 | 0.6 | 0.3 | 0.7 | 0.3 | 0.5 | 0.2 | 0.8 | 0.2 | 0.2 | 0.4 |
| *v*_2_ | 0.6 | 0.2 | 1 | 0 | 0.3 | 0.6 | 0.3 | 0.5 | 0.5 | 0.3 | 0.4 | 0.2 | 0.5 | 0.4 | 0.3 | 0.2 | 0.4 | 0.1 | 0.8 | 0.1 | 0.6 | 0.2 | 0.8 | 0.2 | 0.6 | 0.4 | 0.2 | 0.2 | 0.3 | 0.5 | 0.2 | 0.6 | 0.6 | 0.4 | 0.2 | 0.3 | 0.5 | 0.2 | 0.4 | 0.5 |
| *v*_3_ | 0.6 | 0.4 | 0.4 | 0.5 | 1 | 0 | 0.5 | 0.5 | 0.9 | 0.1 | 0.2 | 0.8 | 0.6 | 0.3 | 0.8 | 0.2 | 0.7 | 0.3 | 0.5 | 0.3 | 0.5 | 0.3 | 0.3 | 0.7 | 0.4 | 0.2 | 0.7 | 0.2 | 0.6 | 0.3 | 0.5 | 0.3 | 0.2 | 0.7 | 0.8 | 0.2 | 0.4 | 0.4 | 0.9 | 0.1 |
| *v*_4_ | 0.3 | 0.4 | 0.5 | 0.3 | 0.3 | 0.4 | 1 | 0 | 0.4 | 0.3 | 0.3 | 0.2 | 0.6 | 0.4 | 0.6 | 0.4 | 0.3 | 0.2 | 0.3 | 0.4 | 0.5 | 0.4 | 0.5 | 0.4 | 0.6 | 0.2 | 0.8 | 0.1 | 0.7 | 0.2 | 0.7 | 0.2 | 0.3 | 0.5 | 0.4 | 0.6 | 0.5 | 0.2 | 0.2 | 0.3 |
| *v*_5_ | 0.5 | 0.3 | 0.6 | 0.2 | 0.6 | 0.4 | 0.5 | 0.2 | 1 | 0 | 0.9 | 0.1 | 0.7 | 0.4 | 0.5 | 0.2 | 0.4 | 0.4 | 0.8 | 0.2 | 0.5 | 0.4 | 0.6 | 0.3 | 0.7 | 0.2 | 0.4 | 0.3 | 0.4 | 0.4 | 0.7 | 0.3 | 0.6 | 0.3 | 0.5 | 0.4 | 0.3 | 0.5 | 0.6 | 0.4 |
| *v*_6_ | 0.9 | 0.2 | 0.4 | 0.3 | 0.5 | 0.5 | 0.3 | 0.6 | 0.3 | 0.7 | 1 | 0 | 0.5 | 0.3 | 0.7 | 0.3 | 0.5 | 0.1 | 0.4 | 0.6 | 0.3 | 0.2 | 0.5 | 0.2 | 0.3 | 0.3 | 0.3 | 0.6 | 0.5 | 0.3 | 0.8 | 0.2 | 0.3 | 0.4 | 0.5 | 0.2 | 0.6 | 0.2 | 0.7 | 0.2 |
| *v*_7_ | 0.3 | 0.6 | 0.7 | 0.3 | 0.8 | 0.2 | 0.7 | 0.3 | 0.8 | 0.2 | 0.2 | 0.7 | 1 | 0 | 0.6 | 0.2 | 0.4 | 0.5 | 0.6 | 0.3 | 0.8 | 0.2 | 0.4 | 0.4 | 0.7 | 0.3 | 0.6 | 0.3 | 0.4 | 0.3 | 0.3 | 0.5 | 0.8 | 0.2 | 0.2 | 0.5 | 0.3 | 0.4 | 0.5 | 0.4 |
| *v*_8_ | 0.5 | 0.3 | 0.8 | 0.2 | 0.9 | 0.1 | 0.6 | 0.2 | 0.2 | 0.7 | 0.7 | 0.3 | 0.4 | 0.4 | 1 | 0 | 0.6 | 0.3 | 0.5 | 0.1 | 0.6 | 0.4 | 0.6 | 0.3 | 0.8 | 0.2 | 0.7 | 0.3 | 0.2 | 0.6 | 0.3 | 0.5 | 0.3 | 0.5 | 0.7 | 0.2 | 0.4 | 0.2 | 0.6 | 0.2 |
| *v*_9_ | 0.7 | 0.2 | 0.6 | 0.2 | 0.6 | 0.3 | 0.3 | 0.6 | 0.6 | 0.4 | 0.8 | 0.2 | 0.4 | 0.5 | 0.8 | 0.2 | 1 | 0 | 0.4 | 0.3 | 0.5 | 0.3 | 0.5 | 0.4 | 0.2 | 0.7 | 0.5 | 0.4 | 0.8 | 0.2 | 0.6 | 0.4 | 0.6 | 0.3 | 0.6 | 0.2 | 0.3 | 0.3 | 0.3 | 0.4 |
| *v*_10_ | 0.5 | 0.4 | 0.3 | 0.6 | 0.5 | 0.2 | 0.3 | 0.7 | 0.3 | 0.5 | 0.3 | 0.5 | 0.5 | 0.4 | 0.5 | 0.5 | 0.7 | 0.2 | 1 | 0 | 0.3 | 0.5 | 0.2 | 0.1 | 0.7 | 0.3 | 0.6 | 0.4 | 0.7 | 0.3 | 0.3 | 0.3 | 0.7 | 0.2 | 0.2 | 0.5 | 0.2 | 0.6 | 0.2 | 0.5 |
| *v*_11_ | 0.4 | 0.2 | 0.6 | 0.4 | 0.7 | 0.3 | 0.9 | 0.1 | 0.4 | 0.6 | 0.6 | 0.3 | 0.8 | 0.2 | 0.6 | 0.1 | 0.4 | 0.4 | 0.3 | 0.3 | 1 | 0 | 0.8 | 0.2 | 0.7 | 0.2 | 0.8 | 0.2 | 0.8 | 0.2 | 0.6 | 0.4 | 0.5 | 0.5 | 0.8 | 0.2 | 0.5 | 0.2 | 0.9 | 0.1 |
| *v*_12_ | 0.7 | 0.2 | 0.3 | 0.4 | 0.3 | 0.7 | 0.7 | 0.3 | 0.2 | 0.6 | 0.3 | 0.2 | 0.8 | 0.2 | 0.2 | 0.6 | 0.2 | 0.5 | 0.4 | 0.4 | 0.5 | 0.4 | 1 | 0 | 0.3 | 0.5 | 0.3 | 0.6 | 0.7 | 0.2 | 0.9 | 0.1 | 0.6 | 0.3 | 0.5 | 0.4 | 0.3 | 0.3 | 0.5 | 0.3 |
| *v*_13_ | 0.4 | 0.3 | 0.7 | 0.3 | 0.7 | 0.3 | 0.8 | 0.2 | 0.4 | 0.2 | 0.7 | 0.2 | 0.6 | 0.3 | 0.4 | 0.2 | 0.3 | 0.4 | 0.3 | 0.3 | 0.8 | 0.2 | 0.5 | 0.4 | 1 | 0 | 0.7 | 0.2 | 0.5 | 0.2 | 0.6 | 0.4 | 0.5 | 0.2 | 0.3 | 0.2 | 0.4 | 0.5 | 0.7 | 0.2 |
| *v*_14_ | 0.4 | 0.6 | 0.6 | 0.2 | 0.6 | 0.4 | 0.6 | 0.3 | 0.5 | 0.2 | 0.2 | 0.3 | 0.3 | 0.4 | 0.5 | 0.3 | 0.6 | 0.3 | 0.5 | 0.4 | 0.3 | 0.4 | 0.4 | 0.1 | 0.4 | 0.2 | 1 | 0 | 0.6 | 0.3 | 0.6 | 0.2 | 0.4 | 0.5 | 0.8 | 0.2 | 0.4 | 0.4 | 0.5 | 0.4 |
| *v*_15_ | 0.2 | 0.4 | 0.3 | 0.7 | 0.5 | 0.5 | 0.2 | 0.3 | 0.8 | 0.2 | 0.6 | 0.2 | 0.5 | 0.3 | 0.8 | 0.2 | 0.5 | 0.4 | 0.3 | 0.5 | 0.5 | 0.4 | 0.3 | 0.7 | 0.5 | 0.4 | 0.8 | 0.2 | 1 | 0 | 0.6 | 0.4 | 0.8 | 0.2 | 0.8 | 0.2 | 0.2 | 0.6 | 0.5 | 0.3 |
| *v*_16_ | 0.7 | 0.2 | 0.7 | 0.3 | 0.4 | 0.3 | 0.7 | 0.2 | 0.4 | 0.6 | 0.4 | 0.4 | 0.5 | 0.2 | 0.7 | 0.3 | 0.7 | 0.2 | 0.3 | 0.3 | 0.6 | 0.3 | 0.3 | 0.5 | 0.7 | 0.1 | 0.5 | 0.3 | 0.5 | 0.3 | 1 | 0 | 0.4 | 0.3 | 0.6 | 0.2 | 0.4 | 0.6 | 0.3 | 0.6 |
| *v*_17_ | 0.3 | 0.4 | 0.6 | 0.2 | 0.4 | 0.5 | 0.4 | 0.6 | 0.8 | 0.2 | 0.5 | 0.3 | 0.7 | 0.3 | 0.8 | 0.2 | 0.4 | 0.5 | 0.6 | 0.4 | 0.3 | 0.2 | 0.7 | 0.2 | 0.4 | 0.2 | 0.6 | 0.3 | 0.3 | 0.5 | 0.2 | 0.7 | 1 | 0 | 0.5 | 0.3 | 0.4 | 0.6 | 0.8 | 0.2 |
| *v*_18_ | 0.6 | 0.3 | 0.3 | 0.3 | 0.7 | 0.2 | 0.6 | 0.4 | 0.4 | 0.6 | 0.3 | 0.2 | 0.8 | 0.2 | 0.5 | 0.5 | 0.4 | 0.3 | 0.5 | 0.2 | 0.4 | 0.5 | 0.5 | 0.4 | 0.7 | 0.3 | 0.8 | 0.1 | 0.6 | 0.2 | 0.3 | 0.2 | 0.3 | 0.3 | 1 | 0 | 0.5 | 0.4 | 0.3 | 0.4 |
| *v*_19_ | 0.4 | 0.2 | 0.8 | 0.2 | 0.8 | 0.2 | 0.4 | 0.6 | 0.4 | 0.4 | 0.6 | 0.4 | 0.6 | 0.3 | 0.4 | 0.4 | 0.8 | 0.2 | 0.4 | 0.4 | 0.5 | 0.2 | 0.8 | 0.2 | 0.3 | 0.4 | 0.7 | 0.2 | 0.8 | 0.2 | 0.3 | 0.5 | 0.7 | 0.2 | 0.7 | 0.2 | 1 | 0 | 0.7 | 0.2 |
| *v*_20_ | 0.6 | 0.4 | 0.4 | 0.5 | 0.6 | 0.3 | 0.7 | 0.2 | 0.3 | 0.6 | 0.7 | 0.2 | 0.8 | 0.1 | 0.3 | 0.6 | 0.6 | 0.3 | 0.3 | 0.6 | 0.7 | 0.3 | 0.5 | 0.3 | 0.5 | 0.2 | 0.9 | 0.1 | 0.4 | 0.3 | 0.5 | 0.3 | 0.4 | 0.3 | 0.9 | 0.1 | 0.5 | 0.5 | 1 | 0 |

**Table S2.** The directed social matrix.

|  | *v*_1_ | *v*_2_ | *v*_3_ | *v*_4_ | *v_5_* | *v*_6_ | *v*_7_ | *v*_8_ | *v*_9_ | *v*_10_ | *v*_11_ | *v*_12_ | *v*_13_ | *v*_14_ | *v*_15_ | *v*_16_ | *v*_17_ | *v*_18_ | *v*_19_ | *v*_20_ |
| --- | --- | --- | --- | --- | --- | --- | --- | --- | --- | --- | --- | --- | --- | --- | --- | --- | --- | --- | --- | --- |
| *v*_1_ | 1 | 0.45 | 0.60 | 0.65 | 0.55 | 0.55 | 0.45 | 0.30 | 0.80 | 0.75 | 0.80 | 0.50 | 0.60 | 0.35 | 0.70 | 0.65 | 0.70 | 0.65 | 0.80 | 0.40 |
| *v*_2_ | 0.70 | 1 | 0.35 | 0.40 | 0.60 | 0.60 | 0.55 | 0.55 | 0.65 | 0.85 | 0.70 | 0.80 | 0.60 | 0.50 | 0.40 | 0.30 | 0.60 | 0.45 | 0.65 | 0.45 |
| *v*_3_ | 0.60 | 0.45 | 1 | 0.50 | 0.90 | 0.20 | 0.65 | 0.80 | 0.70 | 0.60 | 0.60 | 0.30 | 0.60 | 0.75 | 0.65 | 0.60 | 0.25 | 0.80 | 0.50 | 0.90 |
| *v*_4_ | 0.45 | 0.60 | 0.45 | 1 | 0.55 | 0.55 | 0.60 | 0.60 | 0.55 | 0.45 | 0.55 | 0.55 | 0.70 | 0.85 | 0.75 | 0.75 | 0.40 | 0.40 | 0.65 | 0.45 |
| *v*_5_ | 0.60 | 0.70 | 0.60 | 0.65 | 1 | 0.90 | 0.65 | 0.65 | 0.50 | 0.80 | 0.55 | 0.65 | 0.75 | 0.55 | 0.50 | 0.70 | 0.65 | 0.55 | 0.40 | 0.60 |
| *v*_6_ | 0.85 | 0.55 | 0.50 | 0.35 | 0.30 | 1 | 0.60 | 0.70 | 0.70 | 0.40 | 0.55 | 0.65 | 0.50 | 0.35 | 0.60 | 0.80 | 0.45 | 0.65 | 0.70 | 0.75 |
| *v*_7_ | 0.35 | 0.70 | 0.80 | 0.70 | 0.80 | 0.25 | 1 | 0.70 | 0.45 | 0.65 | 0.80 | 0.50 | 0.70 | 0.65 | 0.55 | 0.40 | 0.80 | 0.35 | 0.45 | 0.55 |
| *v*_8_ | 0.60 | 0.80 | 0.90 | 0.70 | 0.25 | 0.70 | 0.50 | 1 | 0.65 | 0.70 | 0.60 | 0.65 | 0.80 | 0.70 | 0.30 | 0.40 | 0.40 | 0.75 | 0.60 | 0.70 |
| *v*_9_ | 0.75 | 0.70 | 0.65 | 0.35 | 0.60 | 0.80 | 0.45 | 0.80 | 1 | 0.55 | 0.60 | 0.55 | 0.25 | 0.55 | 0.80 | 0.60 | 0.65 | 0.70 | 0.50 | 0.45 |
| *v*_10_ | 0.55 | 0.35 | 0.65 | 0.30 | 0.40 | 0.40 | 0.55 | 0.50 | 0.75 | 1 | 0.40 | 0.55 | 0.70 | 0.60 | 0.70 | 0.50 | 0.75 | 0.35 | 0.30 | 0.35 |
| *v*_11_ | 0.60 | 0.60 | 0.70 | 0.90 | 0.40 | 0.65 | 0.80 | 0.75 | 0.50 | 0.50 | 1 | 0.80 | 0.75 | 0.80 | 0.80 | 0.60 | 0.50 | 0.80 | 0.65 | 0.90 |
| *v*_12_ | 0.75 | 0.45 | 0.30 | 0.70 | 0.30 | 0.55 | 0.80 | 0.30 | 0.35 | 0.50 | 0.55 | 1 | 0.40 | 0.35 | 0.75 | 0.90 | 0.65 | 0.55 | 0.50 | 0.60 |
| *v*_13_ | 0.55 | 0.70 | 0.70 | 0.80 | 0.60 | 0.75 | 0.65 | 0.60 | 0.45 | 0.50 | 0.80 | 0.55 | 1 | 0.75 | 0.65 | 0.60 | 0.65 | 0.55 | 0.45 | 0.75 |
| *v*_14_ | 0.40 | 0.70 | 0.60 | 0.65 | 0.65 | 0.45 | 0.45 | 0.60 | 0.65 | 0.55 | 0.45 | 0.65 | 0.60 | 1 | 0.65 | 0.70 | 0.45 | 0.80 | 0.50 | 0.55 |
| *v*_15_ | 0.40 | 0.30 | 0.50 | 0.45 | 0.80 | 0.70 | 0.60 | 0.80 | 0.55 | 0.40 | 0.55 | 0.30 | 0.55 | 0.80 | 1 | 0.60 | 0.80 | 0.80 | 0.30 | 0.60 |
| *v*_16_ | 0.75 | 0.70 | 0.55 | 0.75 | 0.40 | 0.50 | 0.65 | 0.70 | 0.75 | 0.50 | 0.65 | 0.40 | 0.80 | 0.60 | 0.60 | 1 | 0.55 | 0.70 | 0.40 | 0.35 |
| *v*_17_ | 0.45 | 0.70 | 0.45 | 0.40 | 0.80 | 0.60 | 0.70 | 0.80 | 0.45 | 0.60 | 0.55 | 0.75 | 0.60 | 0.65 | 0.40 | 0.25 | 1 | 0.60 | 0.40 | 0.80 |
| *v*_18_ | 0.65 | 0.50 | 0.75 | 0.60 | 0.40 | 0.55 | 0.80 | 0.50 | 0.55 | 0.65 | 0.45 | 0.55 | 0.70 | 0.85 | 0.70 | 0.55 | 0.50 | 1 | 0.55 | 0.45 |
| *v*_19_ | 0.60 | 0.80 | 0.80 | 0.40 | 0.50 | 0.60 | 0.65 | 0.50 | 0.80 | 0.50 | 0.65 | 0.80 | 0.45 | 0.75 | 0.80 | 0.40 | 0.75 | 0.75 | 1 | 0.75 |
| *v*_20_ | 0.60 | 0.45 | 0.65 | 0.75 | 0.35 | 0.75 | 0.85 | 0.35 | 0.65 | 0.35 | 0.70 | 0.60 | 0.65 | 0.90 | 0.55 | 0.60 | 0.55 | 0.90 | 0.50 | 1 |

**Table S3.** The similarity matrix.

|  | *v*_1_ | *v*_2_ | *v*_3_ | *v*_4_ | *v_5_* | *v*_6_ | *v*_7_ | *v*_8_ | *v*_9_ | *v*_10_ | *v*_11_ | *v*_12_ | *v*_13_ | *v*_14_ | *v*_15_ | *v*_16_ | *v*_17_ | *v*_18_ | *v*_19_ | *v*_20_ |
| --- | --- | --- | --- | --- | --- | --- | --- | --- | --- | --- | --- | --- | --- | --- | --- | --- | --- | --- | --- | --- |
| *v*_1_ | 1 | 0.7411 | 0.7455 | 0.7723 | 0.8304 | 0.7768 | 0.7813 | 0.8348 | 0.7679 | 0.7589 | 0.7946 | 0.7946 | 0.8125 | 0.8036 | 0.7500 | 0.7991 | 0.8482 | 0.8214 | 0.7679 | 0.8125 |
| *v*_2_ | 0.7411 | 1 | 0.7455 | 0.7991 | 0.7321 | 0.8036 | 0.7723 | 0.7545 | 0.8125 | 0.7679 | 0.8214 | 0.7857 | 0.8125 | 0.8214 | 0.7232 | 0.7902 | 0.8036 | 0.7946 | 0.7589 | 0.8304 |
| *v*_3_ | 0.7455 | 0.7455 | 1 | 0.7679 | 0.7723 | 0.7634 | 0.8036 | 0.8036 | 0.7902 | 0.8080 | 0.7902 | 0.7902 | 0.7991 | 0.7991 | 0.8348 | 0.7946 | 0.7991 | 0.7902 | 0.8438 | 0.7813 |
| *v*_4_ | 0.7723 | 0.7991 | 0.7679 | 1 | 0.8259 | 0.8259 | 0.8125 | 0.8393 | 0.7991 | 0.7634 | 0.7634 | 0.8170 | 0.8170 | 0.8080 | 0.7813 | 0.7679 | 0.8170 | 0.8080 | 0.7813 | 0.7813 |
| *v*_5_ | 0.8304 | 0.7321 | 0.7723 | 0.8259 | 1 | 0.8125 | 0.7991 | 0.8616 | 0.7500 | 0.7768 | 0.7411 | 0.8304 | 0.8482 | 0.7946 | 0.7768 | 0.7991 | 0.8036 | 0.8125 | 0.7589 | 0.7857 |
| *v*_6_ | 0.7768 | 0.8036 | 0.7634 | 0.8259 | 0.8125 | 1 | 0.7366 | 0.8170 | 0.7679 | 0.7946 | 0.7500 | 0.8482 | 0.8304 | 0.8214 | 0.7411 | 0.8348 | 0.7857 | 0.8661 | 0.7857 | 0.7946 |
| *v*_7_ | 0.7813 | 0.7723 | 0.8036 | 0.8125 | 0.7991 | 0.7366 | 1 | 0.8036 | 0.8259 | 0.8080 | 0.7991 | 0.8080 | 0.8438 | 0.8259 | 0.8080 | 0.7679 | 0.8438 | 0.8348 | 0.8170 | 0.8170 |
| *v*_8_ | 0.8348 | 0.7545 | 0.8036 | 0.8393 | 0.8616 | 0.8170 | 0.8036 | 1 | 0.7723 | 0.7723 | 0.7902 | 0.8080 | 0.8170 | 0.7991 | 0.8259 | 0.7768 | 0.8080 | 0.8348 | 0.7634 | 0.7902 |
| *v*_9_ | 0.7679 | 0.8125 | 0.7902 | 0.7991 | 0.7500 | 0.7679 | 0.8259 | 0.7723 | 1 | 0.7768 | 0.8304 | 0.8304 | 0.8214 | 0.8304 | 0.7857 | 0.7723 | 0.8304 | 0.7946 | 0.7946 | 0.8125 |
| *v*_10_ | 0.7589 | 0.7679 | 0.8080 | 0.7634 | 0.7768 | 0.7946 | 0.8080 | 0.7723 | 0.7768 | 1 | 0.7679 | 0.7768 | 0.8482 | 0.7857 | 0.7946 | 0.8438 | 0.8125 | 0.8482 | 0.8036 | 0.7946 |
| *v*_11_ | 0.7946 | 0.8214 | 0.7902 | 0.7634 | 0.7411 | 0.7500 | 0.7991 | 0.7902 | 0.8304 | 0.7679 | 1 | 0.7857 | 0.8393 | 0.8304 | 0.7589 | 0.8080 | 0.8393 | 0.8036 | 0.7679 | 0.8482 |
| *v*_12_ | 0.7946 | 0.7857 | 0.7902 | 0.8170 | 0.8304 | 0.8482 | 0.8080 | 0.8080 | 0.8304 | 0.7768 | 0.7857 | 1 | 0.8482 | 0.8571 | 0.8125 | 0.8080 | 0.8393 | 0.8304 | 0.8214 | 0.8304 |
| *v*_13_ | 0.8125 | 0.8125 | 0.7991 | 0.8170 | 0.8482 | 0.8304 | 0.8438 | 0.8170 | 0.8214 | 0.8482 | 0.8393 | 0.8482 | 1 | 0.8571 | 0.7857 | 0.8795 | 0.8571 | 0.8571 | 0.8214 | 0.8750 |
| *v*_14_ | 0.8036 | 0.8214 | 0.7991 | 0.8080 | 0.7946 | 0.8214 | 0.8259 | 0.7991 | 0.8304 | 0.7857 | 0.8304 | 0.8571 | 0.8571 | 1 | 0.8036 | 0.8170 | 0.8750 | 0.8393 | 0.8304 | 0.8393 |
| *v*_15_ | 0.7500 | 0.7232 | 0.8348 | 0.7813 | 0.7768 | 0.7411 | 0.8080 | 0.8259 | 0.7857 | 0.7946 | 0.7589 | 0.8125 | 0.7857 | 0.8036 | 1 | 0.7634 | 0.8125 | 0.7768 | 0.7946 | 0.7411 |
| *v*_16_ | 0.7991 | 0.7902 | 0.7946 | 0.7679 | 0.7991 | 0.8348 | 0.7679 | 0.7768 | 0.7723 | 0.8438 | 0.8080 | 0.8080 | 0.8795 | 0.8170 | 0.7634 | 1 | 0.8080 | 0.8438 | 0.7813 | 0.8438 |
| *v*_17_ | 0.8482 | 0.8036 | 0.7991 | 0.8170 | 0.8036 | 0.7857 | 0.8438 | 0.8080 | 0.8304 | 0.8125 | 0.8393 | 0.8393 | 0.8571 | 0.8750 | 0.8125 | 0.8080 | 1 | 0.8482 | 0.8214 | 0.8125 |
| *v*_18_ | 0.8214 | 0.7946 | 0.7902 | 0.8080 | 0.8125 | 0.8661 | 0.8348 | 0.8348 | 0.7946 | 0.8482 | 0.8036 | 0.8304 | 0.8571 | 0.8393 | 0.7768 | 0.8438 | 0.8482 | 1 | 0.7946 | 0.8482 |
| *v*_19_ | 0.7679 | 0.7589 | 0.8438 | 0.7813 | 0.7589 | 0.7857 | 0.8170 | 0.7634 | 0.7946 | 0.8036 | 0.7679 | 0.8214 | 0.8214 | 0.8304 | 0.7946 | 0.7813 | 0.8214 | 0.7946 | 1 | 0.7679 |
| *v*_20_ | 0.8125 | 0.8304 | 0.7813 | 0.7813 | 0.7857 | 0.7946 | 0.8170 | 0.7902 | 0.8125 | 0.7946 | 0.8482 | 0.8304 | 0.8750 | 0.8393 | 0.7411 | 0.8438 | 0.8125 | 0.8482 | 0.7679 | 1 |

**Table S4.** The undirected social matrix.

|  | *v*_1_ | *v*_2_ | *v*_3_ | *v*_4_ | *v_5_* | *v*_6_ | *v*_7_ | *v*_8_ | *v*_9_ | *v*_10_ | *v*_11_ | *v*_12_ | *v*_13_ | *v*_14_ | *v*_15_ | *v*_16_ | *v*_17_ | *v*_18_ | *v*_19_ | *v*_20_ |
| --- | --- | --- | --- | --- | --- | --- | --- | --- | --- | --- | --- | --- | --- | --- | --- | --- | --- | --- | --- | --- |
| *v*_1_ | 1 | 0.45 | 0.60 | 0.45 | 0.55 | 0.55 | 0.35 | 0.30 | 0.75 | 0.55 | 0.60 | 0.50 | 0.55 | 0.35 | 0.40 | 0.65 | 0.45 | 0.65 | 0.60 | 0.40 |
| *v*_2_ | 0.45 | 1 | 0.35 | 0.40 | 0.60 | 0.55 | 0.55 | 0.55 | 0.65 | 0.35 | 0.60 | 0.45 | 0.60 | 0.50 | 0.30 | 0.30 | 0.60 | 0.45 | 0.65 | 0.45 |
| *v*_3_ | 0.60 | 0.35 | 1 | 0.45 | 0.60 | 0.20 | 0.65 | 0.80 | 0.65 | 0.60 | 0.60 | 0.30 | 0.60 | 0.60 | 0.50 | 0.55 | 0.25 | 0.75 | 0.50 | 0.65 |
| *v*_4_ | 0.45 | 0.40 | 0.45 | 1 | 0.55 | 0.35 | 0.60 | 0.60 | 0.35 | 0.30 | 0.55 | 0.55 | 0.70 | 0.65 | 0.45 | 0.75 | 0.40 | 0.40 | 0.40 | 0.45 |
| *v*_5_ | 0.55 | 0.60 | 0.60 | 0.55 | 1 | 0.30 | 0.65 | 0.25 | 0.50 | 0.40 | 0.40 | 0.30 | 0.60 | 0.55 | 0.50 | 0.40 | 0.65 | 0.40 | 0.40 | 0.35 |
| *v*_6_ | 0.55 | 0.55 | 0.20 | 0.35 | 0.30 | 1 | 0.25 | 0.70 | 0.70 | 0.40 | 0.55 | 0.55 | 0.50 | 0.35 | 0.60 | 0.50 | 0.45 | 0.55 | 0.60 | 0.75 |
| *v*_7_ | 0.35 | 0.55 | 0.65 | 0.60 | 0.65 | 0.25 | 1 | 0.50 | 0.45 | 0.55 | 0.80 | 0.50 | 0.65 | 0.45 | 0.55 | 0.40 | 0.70 | 0.35 | 0.45 | 0.55 |
| *v*_8_ | 0.30 | 0.55 | 0.80 | 0.60 | 0.25 | 0.70 | 0.50 | 1 | 0.65 | 0.50 | 0.60 | 0.30 | 0.60 | 0.60 | 0.30 | 0.40 | 0.40 | 0.50 | 0.50 | 0.35 |
| *v*_9_ | 0.75 | 0.65 | 0.65 | 0.35 | 0.50 | 0.70 | 0.45 | 0.65 | 1 | 0.55 | 0.50 | 0.35 | 0.25 | 0.55 | 0.55 | 0.60 | 0.45 | 0.55 | 0.50 | 0.45 |
| *v*_10_ | 0.55 | 0.35 | 0.60 | 0.30 | 0.40 | 0.40 | 0.55 | 0.50 | 0.55 | 1 | 0.40 | 0.50 | 0.50 | 0.55 | 0.40 | 0.50 | 0.60 | 0.35 | 0.30 | 0.35 |
| *v*_11_ | 0.60 | 0.60 | 0.60 | 0.55 | 0.40 | 0.55 | 0.80 | 0.60 | 0.50 | 0.40 | 1 | 0.55 | 0.75 | 0.45 | 0.55 | 0.60 | 0.50 | 0.45 | 0.65 | 0.70 |
| *v*_12_ | 0.50 | 0.45 | 0.30 | 0.55 | 0.30 | 0.55 | 0.50 | 0.30 | 0.35 | 0.50 | 0.55 | 1 | 0.40 | 0.35 | 0.30 | 0.40 | 0.65 | 0.55 | 0.50 | 0.60 |
| *v*_13_ | 0.55 | 0.60 | 0.60 | 0.70 | 0.60 | 0.50 | 0.65 | 0.60 | 0.25 | 0.50 | 0.75 | 0.40 | 1 | 0.60 | 0.55 | 0.60 | 0.60 | 0.55 | 0.45 | 0.65 |
| *v*_14_ | 0.35 | 0.50 | 0.60 | 0.65 | 0.55 | 0.35 | 0.45 | 0.60 | 0.55 | 0.55 | 0.45 | 0.35 | 0.60 | 1 | 0.65 | 0.60 | 0.45 | 0.80 | 0.50 | 0.55 |
| *v*_15_ | 0.40 | 0.30 | 0.50 | 0.45 | 0.50 | 0.60 | 0.55 | 0.30 | 0.55 | 0.40 | 0.55 | 0.30 | 0.55 | 0.65 | 1 | 0.60 | 0.40 | 0.70 | 0.30 | 0.55 |
| *v*_16_ | 0.65 | 0.30 | 0.55 | 0.75 | 0.40 | 0.50 | 0.40 | 0.40 | 0.60 | 0.50 | 0.60 | 0.40 | 0.60 | 0.60 | 0.60 | 1 | 0.25 | 0.55 | 0.40 | 0.35 |
| *v*_17_ | 0.45 | 0.60 | 0.25 | 0.40 | 0.65 | 0.45 | 0.70 | 0.40 | 0.45 | 0.60 | 0.50 | 0.65 | 0.60 | 0.45 | 0.40 | 0.25 | 1 | 0.50 | 0.40 | 0.55 |
| *v*_18_ | 0.65 | 0.45 | 0.75 | 0.40 | 0.40 | 0.55 | 0.35 | 0.50 | 0.55 | 0.35 | 0.45 | 0.55 | 0.55 | 0.80 | 0.70 | 0.55 | 0.50 | 1 | 0.55 | 0.45 |
| *v*_19_ | 0.60 | 0.65 | 0.50 | 0.40 | 0.40 | 0.60 | 0.45 | 0.50 | 0.50 | 0.30 | 0.65 | 0.50 | 0.45 | 0.50 | 0.30 | 0.40 | 0.40 | 0.55 | 1 | 0.50 |
| *v*_20_ | 0.40 | 0.45 | 0.65 | 0.45 | 0.35 | 0.75 | 0.55 | 0.35 | 0.45 | 0.35 | 0.70 | 0.60 | 0.65 | 0.55 | 0.55 | 0.35 | 0.55 | 0.45 | 0.50 | 1 |

**Table S5.** The hybrid trust network matrix.

|  | *v*_1_ | *v*_2_ | *v*_3_ | *v*_4_ | *v_5_* | *v*_6_ | *v*_7_ | *v*_8_ | *v*_9_ | *v*_10_ | *v*_11_ | *v*_12_ | *v*_13_ | *v*_14_ | *v*_15_ | *v*_16_ | *v*_17_ | *v*_18_ | *v*_19_ | | *v*_20_ |
| --- | --- | --- | --- | --- | --- | --- | --- | --- | --- | --- | --- | --- | --- | --- | --- | --- | --- | --- | --- | --- | --- |
| *v*_1_ | 1 | 0.5956 | 0.6728 | 0.6112 | 0.6902 | 0.6634 | 0.5657 | 0.5674 | 0.7590 | 0.6545 | 0.6973 | 0.6473 | 0.6813 | 0.5768 | 0.5750 | 0.7246 | 0.6491 | 0.7357 | 0.6840 | 0.6063 | |
| *v*_2_ | 0.5956 | 1 | 0.5478 | 0.5996 | 0.6661 | 0.6768 | 0.6612 | 0.6523 | 0.7313 | 0.5590 | 0.7107 | 0.6179 | 0.7063 | 0.6607 | 0.5116 | 0.5451 | 0.7018 | 0.6223 | 0.7045 | 0.6402 | |
| *v*_3_ | 0.6728 | 0.5478 | 1 | 0.6090 | 0.6862 | 0.4817 | 0.7268 | 0.8018 | 0.7201 | 0.7040 | 0.6951 | 0.5451 | 0.6996 | 0.6996 | 0.6674 | 0.6723 | 0.5246 | 0.7701 | 0.6719 | 0.7157 | |
| *v*_4_ | 0.6112 | 0.5996 | 0.6090 | 1 | 0.6880 | 0.5880 | 0.7063 | 0.7197 | 0.5746 | 0.5317 | 0.6567 | 0.6835 | 0.7585 | 0.7290 | 0.6157 | 0.7590 | 0.6085 | 0.6040 | 0.5907 | 0.6157 | |
| *v*_5_ | 0.6902 | 0.6661 | 0.6862 | 0.6880 | 1 | 0.5563 | 0.7246 | 0.5558 | 0.6250 | 0.5884 | 0.5706 | 0.5652 | 0.7241 | 0.6723 | 0.6384 | 0.5996 | 0.7268 | 0.6063 | 0.5795 | 0.5679 | |
| *v*_6_ | 0.6634 | 0.6768 | 0.4817 | 0.5880 | 0.5563 | 1 | 0.4933 | 0.7585 | 0.7340 | 0.5973 | 0.6500 | 0.6991 | 0.6652 | 0.5857 | 0.6706 | 0.6674 | 0.6179 | 0.7081 | 0.6929 | 0.7723 | |
| *v*_7_ | 0.5657 | 0.6612 | 0.7268 | 0.7063 | 0.7246 | 0.4933 | 1 | 0.6518 | 0.6380 | 0.6790 | 0.7996 | 0.6540 | 0.7469 | 0.6380 | 0.6790 | 0.5840 | 0.7719 | 0.5924 | 0.6335 | 0.6835 | |
| *v*_8_ | 0.5674 | 0.6523 | 0.8018 | 0.7197 | 0.5558 | 0.7585 | 0.6518 | 1 | 0.7112 | 0.6362 | 0.6951 | 0.5540 | 0.7085 | 0.6996 | 0.5630 | 0.5884 | 0.6040 | 0.6674 | 0.6317 | 0.5701 | |
| *v*_9_ | 0.7590 | 0.7313 | 0.7201 | 0.5746 | 0.6250 | 0.7340 | 0.6380 | 0.7112 | 1 | 0.6634 | 0.6652 | 0.5902 | 0.5357 | 0.6902 | 0.6679 | 0.6862 | 0.6402 | 0.6723 | 0.6473 | 0.6313 | |
| *v*_10_ | 0.6545 | 0.5590 | 0.7040 | 0.5317 | 0.5884 | 0.5973 | 0.6790 | 0.6362 | 0.6634 | 1 | 0.5840 | 0.6384 | 0.6741 | 0.6679 | 0.5973 | 0.6719 | 0.7063 | 0.5991 | 0.5518 | 0.5723 | |
| *v*_11_ | 0.6973 | 0.7107 | 0.6951 | 0.6567 | 0.5706 | 0.6500 | 0.7996 | 0.6951 | 0.6652 | 0.5840 | 1 | 0.6679 | 0.7947 | 0.6402 | 0.6545 | 0.7040 | 0.6697 | 0.6268 | 0.7090 | 0.7741 | |
| *v*_12_ | 0.6473 | 0.6179 | 0.5451 | 0.6835 | 0.5652 | 0.6991 | 0.6540 | 0.5540 | 0.5902 | 0.6384 | 0.6679 | 1 | 0.6241 | 0.6036 | 0.5563 | 0.6040 | 0.7447 | 0.6902 | 0.6607 | 0.7152 | |
| *v*_13_ | 0.6813 | 0.7063 | 0.6996 | 0.7585 | 0.7241 | 0.6652 | 0.7469 | 0.7085 | 0.5357 | 0.6741 | 0.7947 | 0.6241 | 1 | 0.7286 | 0.6679 | 0.7398 | 0.7286 | 0.7036 | 0.6357 | 0.7625 | |
| *v*_14_ | 0.5768 | 0.6607 | 0.6996 | 0.7290 | 0.6723 | 0.5857 | 0.6380 | 0.6996 | 0.6902 | 0.6679 | 0.6402 | 0.6036 | 0.7286 | 1 | 0.7268 | 0.7085 | 0.6625 | 0.8197 | 0.6652 | 0.6947 | |
| *v*_15_ | 0.5750 | 0.5116 | 0.6674 | 0.6157 | 0.6384 | 0.6706 | 0.6790 | 0.5630 | 0.6679 | 0.5973 | 0.6545 | 0.5563 | 0.6679 | 0.7268 | 1 | 0.6817 | 0.6063 | 0.7384 | 0.5473 | 0.6456 | |
| *v*_16_ | 0.7246 | 0.5451 | 0.6723 | 0.7590 | 0.5996 | 0.6674 | 0.5840 | 0.5884 | 0.6862 | 0.6719 | 0.7040 | 0.6040 | 0.7398 | 0.7085 | 0.6817 | 1 | 0.5290 | 0.6969 | 0.5907 | 0.5969 | |
| *v*_17_ | 0.6491 | 0.7018 | 0.5246 | 0.6085 | 0.7268 | 0.6179 | 0.7719 | 0.6040 | 0.6402 | 0.7063 | 0.6697 | 0.7447 | 0.7286 | 0.6625 | 0.6063 | 0.5290 | 1 | 0.6741 | 0.6107 | 0.6813 | |
| *v*_18_ | 0.7357 | 0.6223 | 0.7701 | 0.6040 | 0.6063 | 0.7081 | 0.5924 | 0.6674 | 0.6723 | 0.5991 | 0.6268 | 0.6902 | 0.7036 | 0.8197 | 0.7384 | 0.6969 | 0.6741 | 1 | 0.6723 | 0.6491 | |
| *v*_19_ | 0.6840 | 0.7045 | 0.6719 | 0.5907 | 0.5795 | 0.6929 | 0.6335 | 0.6317 | 0.6473 | 0.5518 | 0.7090 | 0.6607 | 0.6357 | 0.6652 | 0.5473 | 0.5907 | 0.6107 | 0.6723 | 1 | 0.6340 | |
| *v*_20_ | 0.6063 | 0.6402 | 0.7157 | 0.6157 | 0.5679 | 0.7723 | 0.6835 | 0.5701 | 0.6313 | 0.5723 | 0.7741 | 0.7152 | 0.7625 | 0.6947 | 0.6456 | 0.5969 | 0.6813 | 0.6491 | 0.6340 | 1 | |

**Table S6.** The updated hybrid trust network matrix.

|  | *v*_1_ | *v*_2_ | *v*_3_ | *v*_4_ | *v_5_* | *v*_6_ | *v*_7_ | *v*_8_ | *v*_9_ | *v*_10_ | *v*_11_ | *v*_12_ | *v*_13_ | *v*_14_ | *v*_15_ | *v*_16_ | *v*_17_ | *v*_18_ | *v*_19_ | *v*_20_ |
| --- | --- | --- | --- | --- | --- | --- | --- | --- | --- | --- | --- | --- | --- | --- | --- | --- | --- | --- | --- | --- |
| *v*_1_ | 1 | 0.6441 | 0.7538 | 0.6616 | 0.7244 | 0.7179 | 0.6248 | 0.5880 | 0.8234 | 0.7250 | 0.7213 | 0.6946 | 0.7159 | 0.6144 | 0.6428 | 0.7677 | 0.6771 | 0.7677 | 0.7203 | 0.6469 |
| *v*_2_ | 0.6441 | 1 | 0.5827 | 0.6183 | 0.7154 | 0.7207 | 0.6890 | 0.6989 | 0.7726 | 0.5801 | 0.9446 | 0.6670 | 0.7270 | 0.6740 | 0.5597 | 0.5649 | 0.7183 | 0.6448 | 0.9649 | 0.6699 |
| *v*_3_ | 0.7538 | 0.5827 | 1 | 0.8228 | 0.9816 | 0.5717 | 0.9840 | 0.9184 | 0.7905 | 0.9813 | 0.7196 | 0.6210 | 0.8645 | 0.8627 | 0.8396 | 0.8498 | 0.9759 | 0.9048 | 0.6782 | 0.7868 |
| *v*_4_ | 0.6616 | 0.6183 | 0.8228 | 1 | 0.8524 | 0.7477 | 0.8627 | 0.9808 | 0.7414 | 0.7780 | 0.6965 | 0.8108 | 0.9825 | 0.9773 | 0.9621 | 0.9843 | 0.8110 | 0.9732 | 0.6290 | 0.7770 |
| *v*_5_ | 0.7244 | 0.7154 | 0.9816 | 0.8524 | 1 | 0.6317 | 0.9874 | 0.7710 | 0.7300 | 0.9742 | 0.6030 | 0.6336 | 0.8665 | 0.8495 | 0.8396 | 0.8111 | 0.9906 | 0.8114 | 0.6172 | 0.6564 |
| *v*_6_ | 0.7179 | 0.7207 | 0.5717 | 0.7477 | 0.6317 | 1 | 0.6015 | 0.8655 | 0.9820 | 0.6784 | 0.7175 | 0.9850 | 0.8000 | 0.7565 | 0.8247 | 0.7985 | 0.7006 | 0.8150 | 0.7493 | 0.9890 |
| *v*_7_ | 0.6248 | 0.6890 | 0.9840 | 0.8627 | 0.9874 | 0.6015 | 1 | 0.8350 | 0.7032 | 0.9812 | 0.8274 | 0.7209 | 0.8773 | 0.8185 | 0.8484 | 0.8068 | 0.9924 | 0.7937 | 0.6445 | 0.7445 |
| *v*_8_ | 0.5880 | 0.6989 | 0.9184 | 0.9808 | 0.7710 | 0.8655 | 0.8350 | 1 | 0.8444 | 0.8329 | 0.7249 | 0.7241 | 0.9931 | 0.9859 | 0.9684 | 0.9926 | 0.8103 | 1.0000 | 0.6818 | 0.7420 |
| *v*_9_ | 0.8234 | 0.7726 | 0.7905 | 0.7414 | 0.7300 | 0.9820 | 0.7032 | 0.8444 | 1 | 0.7530 | 0.6960 | 0.9755 | 0.7091 | 0.8157 | 0.8031 | 0.8269 | 0.7075 | 0.8098 | 0.7016 | 0.9795 |
| *v*_10_ | 0.7250 | 0.5801 | 0.9813 | 0.7780 | 0.9742 | 0.6784 | 0.9812 | 0.8329 | 0.7530 | 1 | 0.6070 | 0.7253 | 0.8344 | 0.8445 | 0.8065 | 0.8327 | 0.9863 | 0.7912 | 0.5676 | 0.6564 |
| *v*_11_ | 0.7213 | 0.9446 | 0.7196 | 0.6965 | 0.6030 | 0.7175 | 0.8274 | 0.7249 | 0.6960 | 0.6070 | 1 | 0.7163 | 0.8067 | 0.6450 | 0.6963 | 0.7256 | 0.6623 | 0.6436 | 0.9318 | 0.7960 |
| *v*_12_ | 0.6946 | 0.6670 | 0.6210 | 0.8108 | 0.6336 | 0.9850 | 0.7209 | 0.7241 | 0.9755 | 0.7253 | 0.7163 | 1 | 0.7611 | 0.7503 | 0.7147 | 0.7583 | 0.7941 | 0.8103 | 0.6967 | 0.9886 |
| *v*_13_ | 0.7159 | 0.7270 | 0.8645 | 0.9825 | 0.8665 | 0.8000 | 0.8773 | 0.9931 | 0.7091 | 0.8344 | 0.8067 | 0.7611 | 1 | 0.9845 | 0.9736 | 0.9935 | 0.8657 | 0.9924 | 0.6571 | 0.8468 |
| *v*_14_ | 0.6144 | 0.6740 | 0.8627 | 0.9773 | 0.8495 | 0.7565 | 0.8185 | 0.9859 | 0.8157 | 0.8445 | 0.6450 | 0.7503 | 0.9845 | 1 | 0.9782 | 0.9846 | 0.8210 | 0.9915 | 0.6834 | 0.8174 |
| *v*_15_ | 0.6428 | 0.5597 | 0.8396 | 0.9621 | 0.8396 | 0.8247 | 0.8484 | 0.9684 | 0.8031 | 0.8065 | 0.6963 | 0.7147 | 0.9736 | 0.9782 | 1 | 0.9775 | 0.8111 | 0.9842 | 0.5738 | 0.8043 |
| *v*_16_ | 0.7677 | 0.5649 | 0.8498 | 0.9843 | 0.8111 | 0.7985 | 0.8068 | 0.9926 | 0.8269 | 0.8327 | 0.7256 | 0.7583 | 0.9935 | 0.9846 | 0.9775 | 1 | 0.7693 | 0.9942 | 0.6305 | 0.7417 |
| *v*_17_ | 0.6771 | 0.7183 | 0.9759 | 0.8110 | 0.9906 | 0.7006 | 0.9924 | 0.8103 | 0.7075 | 0.9863 | 0.6623 | 0.7941 | 0.8657 | 0.8210 | 0.8111 | 0.7693 | 1 | 0.8374 | 0.6210 | 0.7484 |
| *v*_18_ | 0.7677 | 0.6448 | 0.9048 | 0.9732 | 0.8114 | 0.8150 | 0.7937 | 1.0000 | 0.8098 | 0.7912 | 0.6436 | 0.8103 | 0.9924 | 0.9915 | 0.9842 | 0.9942 | 0.8374 | 1 | 0.7081 | 0.7764 |
| *v*_19_ | 0.7203 | 0.9649 | 0.6782 | 0.6290 | 0.6172 | 0.7493 | 0.6445 | 0.6818 | 0.7016 | 0.5676 | 0.9318 | 0.6967 | 0.6571 | 0.6834 | 0.5738 | 0.6305 | 0.6210 | 0.7081 | 1 | 0.6995 |
| *v*_20_ | 0.6469 | 0.6699 | 0.7868 | 0.7770 | 0.6564 | 0.9890 | 0.7445 | 0.7420 | 0.9795 | 0.6564 | 0.7960 | 0.9886 | 0.8468 | 0.8174 | 0.8043 | 0.7417 | 0.7484 | 0.7764 | 0.6995 | 1 |
